# Global, regional, and national cancer incidence and death for 29 cancer groups in 2019 and trends analysis of the global cancer burden, 1990–2019

**DOI:** 10.1186/s13045-021-01213-z

**Published:** 2021-11-22

**Authors:** Longfei Lin, Zhiyong Li, Lei Yan, Yuling Liu, Hongjun Yang, Hui Li

**Affiliations:** 1 Institute Chinese Materia Medica China Academy of Chinese Medical Sciences, Beijing, China; 2 Fengtai District Community Health Center, Beijing, China; 3 Experimental Medical Center, China Academy of Chinese Medical Sciences, Beijing, China

**Keywords:** Global burden of disease, Cancer, Incidence, Death

## Abstract

**Background and aims:**

Cancer will soon become the leading cause of death in every country in the twenty-first century. This study aimed to analyze the mortality and morbidity of 29 types of cancer in 204 countries or regions from 1990 to 2019 to guide global cancer prevention and control.

**Methods:**

Detailed information for 29 cancer groups was collected from the Global Burden of Disease Study in 2019. The age-standardized incidence rate (ASIR) and age-standardized death rate (ASDR) of the 29 cancer groups were calculated based on sex, age, region, and country. In addition, separate analyses were performed for major cancer types.

**Results:**

In 2019, more than 10 million people died from cancer, which was approximately twice the number in 1990. Tracheal, bronchus, and lung (TBL) cancers collectively showed the highest death rate, and the ASDR of pancreatic cancer increased by 24%, which was cancer with the highest case fatality rate (CFR). The global cancer ASIR showed an increasing trend, with testicular cancer, thyroid cancer, and malignant skin melanoma showing a significant increase. The ASDR and ASIR of cancer in males were about 1.5 times higher than that in females. Individuals over 50 years had the highest risk of developing cancer, with incidences and deaths in this age group accounting for more than 85% of cancers in all age groups. Asia has the heaviest cancer burden due to its high population density, with esophageal cancer in this region accounting for 53% of the total fatalities related to this type of cancer in the world. In addition, the mortality and morbidity of most cancers increased with the increase in the development or socio-demographic index (SDI) in the SDI regions based on the World Bank's Human Development Index (HDI), with cancer characteristics varying in the different countries globally.

**Conclusions:**

The global cancer burden continues to increase, with substantial mortality and morbidity differences among the different regions, ages, countries, gender, and cancer types. Effective and locally tailored cancer prevention and control measures are essential in reducing the global cancer burden in the future.

**Supplementary Information:**

The online version contains supplementary material available at 10.1186/s13045-021-01213-z.

## Introduction

According to the World Health Organization (WHO) statistics, in 2019, cancer ranks as the first or second leading cause of death in 112 countries globally and third or fourth in another 23 countries [1]. In countries with higher economic development levels, mortality from stroke and coronary heart disease are decreasing, while cancer is becoming the main and only disease hindering life expectancy [2]. Due to population growth and aging, a significant downward trend of the main high-risk cancers has not been seen yet, and their burden has increased rapidly in some countries. This reflects the changes in the prevalence and distribution of main risk factors related to social and economic development [3, 4].

Presently, cancer morbidity and mortality differences exist among regions and countries, mainly due to the difference in population risk factors caused by social-economic changes. Analysis of cancers in different populations and economic regions worldwide can help peers in clinical and related research fields and health administrative institutions formulate relevant measures for cancer prevention and control. This study describes the global burden of cancer using results from the Global Burden of Disease (GBD) 2019 study for 29 cancer groups by gender and age for 204 countries and regions [5]. Specifically, this study analyzed the global burden of cancer in 2019, including the specific cancers, such as cancer with the highest case fatality rate (CFR) and mortality, the highest burden in females, the highest cancer in children, the most geographically differentiated cancer, and the CFR of different types of cancer, genders, and regions. The findings in this study will provide insights into global cancer prevention and control.

## Methods

### Data source

The types of cancer were assigned into 29 cancer groups according to the International Classification of Diseases (ICD). The annual incidence, death number, and age-standardized rate of the 29 cancer types in different genders, regions, countries, and ages were collected from the GBD database. Data from 204 countries were collected. Further, these countries were divided into five regions (low, low-medium, medium, high-medium, and high level) based on the socio-demographic index (SDI). In addition, the global social-economic development levels were divided into four levels: high, upper-middle, lower-middle, and low level based on the HDI. Based on geography, the world was divided into 21 regions (Table 1). Morbidity and mortality in these regions were calculated to unearth the trends in the epidemiology of malignant tumors in the regional environment, national ethnicity, and living habits. The cancer incidence, mortality, and morbidity were determined using estimates from individual cancer registries or aggregated databases, including the Cancer Incidence in Five Continents (CI5), EUREG, and NORDCAN. Since most cancer registries only had reports on cancer incidence and where data on mortality for some locations and time points were scarce, mortality was estimated from the cancer incidence using separately modeled mortality-to-incidence ratios (MIR). The uncertainty interval (UI) was set at 95%. The GBD estimates calculations were made 1,000 times to determine the UI, using distribution samples rather than point estimates for data inputs, data transformations, and model choice. The 95^th^ uncertainty interval was determined by the 25^th^ and 975^th^ value of the 1,000 values after ordering them from smallest to largest. Larger uncertainty intervals result from limited data availability, small studies, and conflicting data, while smaller uncertainty intervals result from extensive data availability, large studies, and consistent data across sources. Additional metadata from each source are available in the online GBD citation tool, http://ghdx.healthdata.org/gbd-2019 [6–8].

**Table 1:** The incidence number, ASIR, death number, ASDR and case fatality rate of 29 cancer types in 1990 and 2019

Cancers	1990					2019				
	ASDR	Death number	ASIR	Incidence number	Case fatality rate(%)	ASDR	Death number	ASIR	Incidence number	Case fatality rate (%)
Esophageal cancer	8.18 (8.97–6.4)	319,332 (350,802–248,666)	8.06 (8.83–6.41)	319,969 (351,210–253,395)	101.5	6.11 (6.76–5.38)	498,067 (551,462–438,411)	6.51 (7.25–5.69)	534,563 (595,342–466,513)	93.9
Stomach cancer	20.48 (21.62–19.25)	788,317 (833,999–742,787)	22.44 (23.59–21.21)	883,396 (929,174–834,237)	91.3	11.88 (12.82–10.82)	957,185 (1,034,646–870,949)	15.59 (17.15–14.11)	1,269,806 (1,399,817–1,150,487)	76.2
Liver cancer	8.93 (9.9–8.09)	365,215 (405,774–329,967)	8.98 (9.97–8.1)	373,390 (415,748–335,890)	99.4	5.95 (6.44–5.44)	484,577 (525,798–444,091)	6.51 (7.16–5.95)	534,364 (588,639–486,550)	91.4
Larynx cancer	2.19 (2.29–2.08)	87,459 (91,551–83,182)	3.06 (3.17–2.93)	124,643 (129,440–119,744)	71.6	1.49 (1.61–1.39)	123,356 (132,798–114,941)	2.51 (2.69–2.32)	209,149 (224,620–193,876)	59.4
Tracheal, bronchus, and lung cancer	27.3 (28.59–26.03)	1,065,139 (1,117,181–1,019,217)	28.39 (29.67–27.18)	1,124,006 (1,176,491–1,077,618)	96.2	25.18 (27.01–23.16)	2,042,640 (2,193,269–1,879,241)	27.66 (29.99–25.28)	2,259,998 (2,451,832–2,067,316)	91.0
Breast cancer	9.8 (10.21–9.3)	380,905 (396,714–364,815)	21.44 (22.1–20.65)	876,993 (903,815–849,689)	45.7	8.62 (9.25–7.95)	700,660 (751,555–647,384)	24.17 (26.24–22.11)	2,002,354 (2,172,540–1,832,150)	35.7
Cervical cancer	4.46 (5.31–4)	184,527 (218,942–164,836)	7.64 (9.01–6.87)	335,642 (393,893–300,354)	58.4	3.4 (3.81–2.9)	280,479 (313,930–238,864)	6.81 (7.66–5.81)	565,541 (636,435–481,524)	49.9
Uterine cancer	1.48 (1.58–1.35)	56,130 (60,199–51,104)	4.61 (4.82–4.31)	187,191 (196,030–174,631)	32.1	1.13 (1.25–1.01)	91,641 (101,502–82,389)	5.21 (5.75–4.75)	435,041 (479,729–397,021)	21.7
Prostate cancer	6.98 (8.1–5.69)	232,999 (268,882–191,398)	14.25 (16.62–11.18)	524,110 (613,006–409,133)	49.0	6.32 (7.71–5.42)	486,836 (593,689–420,498)	17.39 (22.5–15.12)	1,410,452 (1,825,766–1,227,900)	36.3
Colon and rectum cancer	14.31 (14.88–13.52)	518,126 (537,877–493,682)	22.25 (22.97–21.29)	842,098 (868,574–810,408)	64.3	13.69 (14.51–12.6)	1,085,797 (1,149,679–1,002,795)	26.71 (28.89–24.58)	2,166,168 (2,342,842–1,996,298)	51.3
Lip and oral cavity cancer	2.44 (2.6–2.28)	96,628 (103,050–90,592)	4.28 (4.51–4.07)	175,626 (184,913–167,516)	57.0	2.44 (2.66–2.22)	199,398 (218,059–181,651)	4.52 (4.89–4.13)	373,098 (403,866–340,884)	54.0
Nasopharynx cancer	1.26 (1.36–1.15)	53,459 (57,906–48,875)	1.55 (1.67–1.42)	67,518 (72,995–61,729)	81.3	0.86 (0.93–0.79)	71,610 (77,625–65,442)	2.12 (2.4–1.87)	176,502 (199,917–156,046)	40.6
Other pharynx cancer	1.25 (1.37–1.17)	51,460 (56,457–47,974)	1.6 (1.72–1.5)	66,531 (71,527–62,626)	78.1	1.37 (1.51–1.24)	114,207 (126,039–103,154)	1.99 (2.15–1.83)	166,901 (180,359–152,960)	68.8
Gallbladder and biliary tract cancer	2.6 (2.93–2.3)	94,855 (107,240–85,139)	2.91 (3.22–2.58)	107,787 (119,860–96,900)	89.3	2.17 (2.38–1.81)	172,441 (188,615–144,899)	2.49 (2.75–2.09)	199,211 (219,615–166,769)	87.1
Pancreatic cancer	5.34 (5.52–5.07)	198,051 (204,763–189,329)	5.22 (5.4–4.97)	197,348 (203,971–188,604)	102.3	6.62 (7.06–6.11)	531,107 (566,537–491,948)	6.57 (7.09–6)	530,297 (573,635–486,175)	100.8
Malignant skin melanoma	0.85 (1.1–0.72)	33,083 (43,094–27,827)	2.56 (3.25–2.05)	107,380 (134,056–85,128)	33.2	0.79 (0.89–0.58)	62,844 (71,001–46,317)	3.56 (4.19–2.63)	289,953 (341,965–214,481)	22.2
Non-melanoma skin cancer	0.69 (0.73–0.63)	23,222 (24,436–21,441)	54.08 (62.08–46.97)	1,951,299 (2,237,075–1,692,794)	1.3	0.73 (0.78–0.65)	56,054 (59,792–50,415)	79.1 (86.63–72.29)	6,353,687 (6,952,145–5,805,441)	0.9
Ovarian cancer	2.5 (2.8–2.32)	97,363 (109,761–89,703)	3.42 (3.84–3.17)	141,706 (160,779–130,541)	73.1	2.43 (2.66–2.15)	198,412 (217,665–175,357)	3.58 (4–3.17)	294,422 (329,727–260,649)	67.9
Testicular cancer	0.15 (0.16–0.14)	7224 (7906–6792)	0.96 (1.12–0.78)	51,893 (61,388–41,490)	15.6	0.14 (0.15–0.13)	10,842 (11,902–9961)	1.4 (1.67–1.18)	109,313 (129,454–93,372)	10.0
Kidney cancer	1.86 (1.93–1.77)	72,100 (74,674–68,876)	3.53 (3.63–3.4)	145,906 (150,333–140,803)	52.7	2.08 (2.2–1.93)	166,438 (176,302–155,461)	4.55 (4.93–4.22)	371,747 (402,350–344,594)	45.7
Bladder cancer	3.49 (3.66–3.27)	121,500 (127,171–114,751)	6.27 (6.5–5.98)	234,754 (243,075–225,464)	55.7	2.94 (3.13–2.7)	228,735 (243,193–210,743)	6.52 (7.09–5.93)	524,305 (569,434–475,952)	45.1
Brain and central nervous system cancer	3.08 (4.01–2.71)	139,632 (182,291–119,905)	3.82 (5–3.34)	179,051 (237,072–152,530)	80.6	3.05 (3.36–2.29)	246,253 (270,930–185,642)	4.34 (4.86–3.27)	347,992 (388,896–262,084)	70.3
Thyroid cancer	0.6 (0.66–0.56)	22,966 (25,228–21,554)	2.01 (2.12–1.9)	87,583 (92,717–82,236)	29.9	0.57 (0.61–0.51)	45,576 (48,775–41,290)	2.83 (3.06–2.56)	233,847 (252,807–211,637)	20.1
Mesothelioma	0.4 (0.44–0.36)	15,385 (17,017–13,815)	0.49 (0.54–0.44)	19,072 (21,157–17,055)	81.6	0.36 (0.39–0.33)	29,251 (31,006–26,668)	0.43 (0.47–0.38)	34,511 (37,771–31,199)	83.7
Hodgkin lymphoma	0.61 (0.66–0.48)	27,602 (30,235–21,663)	1.26 (1.35–1.02)	59,688 (64,237–48,254)	48.4	0.34 (0.4–0.29)	27,552 (31,813–23,685)	1.1 (1.27–0.98)	87,509 (101,425–77,941)	30.9
Non-Hodgkin lymphoma	3.15 (3.29–3)	126,082 (131,976–119,768)	4.65 (4.93–4.37)	190,725 (203,617–179,033)	67.7	3.19 (3.39–2.98)	254,614 (270,353–237,714)	5.73 (6.25–5.21)	457,077 (498,785–416,894)	55.7
Multiple myeloma	1.4 (1.58–1.28)	51,862 (58,980–47,710)	1.73 (1.93–1.59)	65,941 (74,059–60,780)	80.9	1.42 (1.52–1.24)	113,474 (121,735–99,527)	1.92 (2.12–1.68)	155,688 (172,577–136,585)	74.0
Leukemia	5.82 (6.44–5.25)	263,263 (298,696–233,664)	9.6 (11.02–8.14)	474,924 (560,550–388,559)	60.6	4.26 (4.58–3.91)	334,592 (360,214–306,818)	8.22 (8.94–7.5)	643,579 (699,729–586,980)	51.8
Other malignant neoplasms	5.64 (6.02–5.08)	238,783 (255,399–215,826)	8.49 (9.05–7.73)	379,732 (407,125–347,417)	66.4	5.14 (5.58–4.48)	408,167 (443,578–355,053)	10.45 (11.38–9.33)	831,446 (905,631–741,237)	49.2

For the first time, the GBD 2019 provided an independent estimate of the population in 204 countries and territories, using a standardized and replicable approach, which provided a comprehensive update on fertility and migration. In addition, the GBD 2019 produced mortality and life expectancy estimates for a total of 990 locations at the most detailed level. A total of 1250 censuses and 747 location-years of population registry data were identified. The Bayesian population model was used to reconciles censuses and registry data based on population size consistent with the GBD fertility and mortality estimates [9, 10].

The GBD estimation method has significant benefits. For example, terminally ill cancer patients in low- and middle-income countries fill out the cancer registry where the patients die before getting attention from health care providers. In such cases, GBD accurately estimates the cancer mortality that other databases such as CI5 and GLOBOCAN might have missed. Thus, GBD estimates accurately reflect the overall cancer burden.

### Statistical analysis

We used age-standardized incidence rates (ASIR) and age-standardized death rates (ASDR) to quantify regional morbidity and mortality trends in the 29 cancer types. The age-standardized rate (per 100,000 population) was calculated by the direct method. Standardization was crucial in this study as it eliminates the bias when comparing proportions or rates. For example, it eliminates the influence of gender, age, etc., between two groups, allowing the analysis of substantive differences. The case fatality rate (CFR) was calculated by dividing the mortality rate with the morbidity rate of the cancers, i.e.,

$${\text{CFR}} = \frac{{{\text{Mortality }}\;{\text{rate}}}}{{{\text{Morbidity }}\;{\text{rate}}}} \times 100$$

Global cancer morbidity and mortality from 1990 to 2019 were calculated using the Joinpoint regression model. The National Cancer Institute (NCI) Joinpoint regression program software (version 4.9.0.0) was used to conduct change-trend analyses and identify statistically significant differences, which were used in calculating the annual percentage change (APC) for each trend phase.

## Result

### General status of global cancer burden in 2019

The incidence, ASIR, death number, and ASDR in the 29 cancer types from 1990 to 2019 are shown in Table 1. In 2019, more than 10 million people died from cancer, which was twice the number in 1990. Besides, 23 million had cancer in 2019, which was more than twice the number in 1990. Nevertheless, from 1990, the ASDR showed a downward trend, while the ASIR showed an upward trend. Based on the 2019 cancer data, cancers with the highest mortality rate were tracheal, bronchus, and lung (TBL) cancer, colon and rectum cancer, and stomach cancer, with ASDR of 25.18 (27.01–23.16), 13.69 (14.51–12.6) and 11.88 (12.82–10.82), respectively. The number of deaths attributed to these cancers were 2,042,640 (2,193,269–1,879,241), 1,085,797 (1,149,679–1,002,795) and 957,185 (1,034,646–870,949), respectively, which accounted for 40.8% of all cancer deaths in 2019. In addition, breast, pancreatic, prostate, esophageal and liver cancers had an ASDR higher than 5. TBL cancer recorded the highest death rate in 2019, almost twice the rate in 1990. Although the ASDR of stomach cancer was relatively high, it has decreased by nearly half compared to 1990, but its associated deaths have increased by about 20%. In contrast, the mortality rate of various cancers has decreased, with Hodgkin lymphoma achieving the most significant decrease (44%), from 0.61 (0.66–0.48) to 0.34 (0.4–0.29) when compared to their rates in 1990. In addition, the ASDR of liver cancer, larynx cancer, and nasopharynx cancer decreased by more than 30%. However, the ASDR of a few cancers showed an increasing trend, such as pancreatic cancer [5.34 (5.52–5.07) to 6.62 (7.06–6.11)], with an increase of 24% and kidney cancer [1.86 (1.93–1.77) to 2.08 (2.2–1.93)], which increased by 11.8%. The ASDR of pharynx cancer, non-melanoma skin cancer, non-Hodgkin lymphoma, and multiple myeloma also showed a slight increase.

Non-melanoma skin cancer, TBL cancer, colon and rectum cancer, breast cancer, prostate cancer, stomach cancer, and other malignant neoplasms had the highest morbidity rate in 2019. The ASIR of these cancers were 79.1 (86.63 72.29), 27.66 (29.99 25.28), 26.71 (28.89 24.58), 24.17 (26.24 22.11), 17.39 (22.5 15.12), 15.59 (17.15 14.11), and 10.45 (11.38 9.33), respectively. The incidence of these cancers were 6,353,687 (6,952,145–5,805,441), 2,259,998 (2,451,832–2,067,316), 2,166,168 (2,342,842–1,996,298), 2,002,354 (2,172,540–1,832,150), 1,410,452 (1, 825,766–1,227,900), 1,269,806 (1,399,817–1,150,487), and 831,446 (905,631–741,237), respectively. Compared to 1990, the ASIR increased significantly in non-melanoma skin cancer (46.3%), testicular cancer (45.8%), thyroid cancer (40.8%), malignant skin cancer (39.1%), and nasopharynx cancer (36.8%). Although the collective ASIR showed an increasing trend, the ASIR of storm cancer, liver cancer, esophageal cancer and larynx cancer, decreased by 30.5%, 27.5%, 19.2% and 18.0%, respectively.

Besides, pancreatic cancer, esophageal cancer, liver cancer, TBL cancer, gallbladder, and biliary tract cancer, and mesothelioma had the highest CFR in 2019 with 100.8%, 93.9%, 91.4%, 91.0%, 87.2%, 83.7%, respectively. Cancers with the lowest CFR were non-melanoma skin cancer (0.9%), testicular cancer (10.0%), thyroid cancer (20.1%), uterine cancer (21.7%), and malignant skin cancer (22.2%). In contrast to 1990, the CFR of nasopharynx cancer, uterine cancer, Hodgkin lymphoma, testicular cancer, malignant skin melanoma, and thyroid cancer was decreased by over 30%. However, the CFR of mesothelioma, pancreatic, esophageal, liver, TBL, gallbladder, and biliary tract cancers, decreased by approximately 10%.

### Cancer burden influenced by gender and age factors

**Fig. 1:**
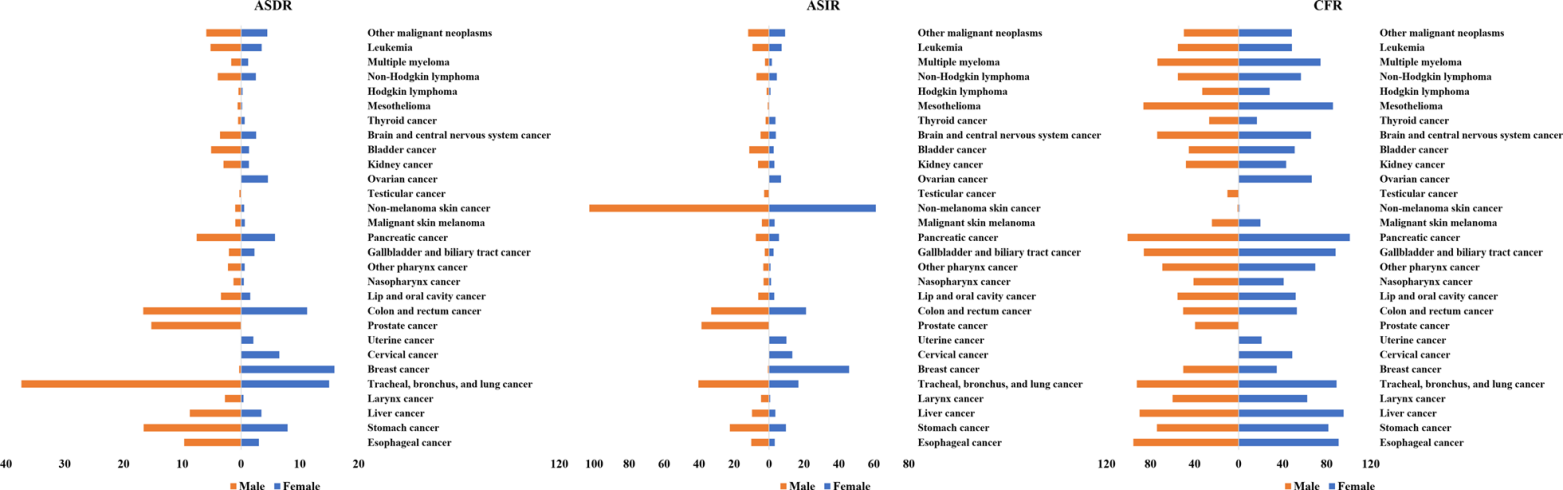
The age-standardized global cancer deaths and incidence, and case fatality rate (CFR) of 29 specified groups in 2019 by gender

With respect to gender, the morbidity and mortality of cancer in men were 1.5 times higher than in women (Fig. 1). In 2019, more than 5.5 million men and 4.3 million women died from cancer. In males, TBL cancer recorded the highest ASDR [37.38 (40.74–34.09)], with 1,386,094 (1,513,800–1,260,237) fatalities, and this accounted for 24.4% of total fatalities in men. Other cancers that recorded a high ASDR in males were colon and rectum cancer [16.64 (17.85–15.39)], stomach cancer [16.59 (18.34–14.80)], prostate cancer [15.28 (18.57–13.00)], and esophageal cancer [9.68(10.96–8.34)]. Besides, these cancers demonstrated a significantly high ASIR in males with 40.44 (44.42–36.55), 33.06 (6.15–30.22), 22.39 (25.34–19.80), 38.63 (49.83–33.63) and 10.13 (11.56–8.73), respectively. In females, the ASDR of breast cancer was the highest [15.88 (17.07–14.66)], with 688,562 (739,571–635,323) deaths, and this accounted for 15.9% of total deaths associated with cancer in women. The ASDR of TBL cancer [14.99 (16.41–13.48)], colon and rectum cancer [11.24 (12.17–10.01)], stomach cancer [7.92 (8.76–7.07)], and cervical cancer [6.51 (7.29–5.55)] were next to breast cancer in females.

The mortality and morbidity of the common cancers in males and females were also significantly different. For example, the ASDR of larynx cancer in males 2.74 (2.98–2.54) was six times higher than that in females (0.41 (0.45–0.37). In addition, the mortality of esophageal cancer, other pharynx cancer, bladder cancer, and mesothelioma in males was over three times that of females. Except for breast cancer, the ASDR of the gallbladder and biliary tract cancer and thyroid cancer was also higher in females than males. In terms of CFR, pancreatic cancer was the highest in both males and females. The CFR of stomach cancer and liver cancer in females was 81.6 and 95.4, slightly higher than those of males. Although the morbidity [0.65 (0.72–0.58)] and mortality [0.33 (0.36–0.29)] of breast cancer were lower in males, they recorded a significantly high CFR (50.3%) compared to females (34.6%).

**Fig. 2:**
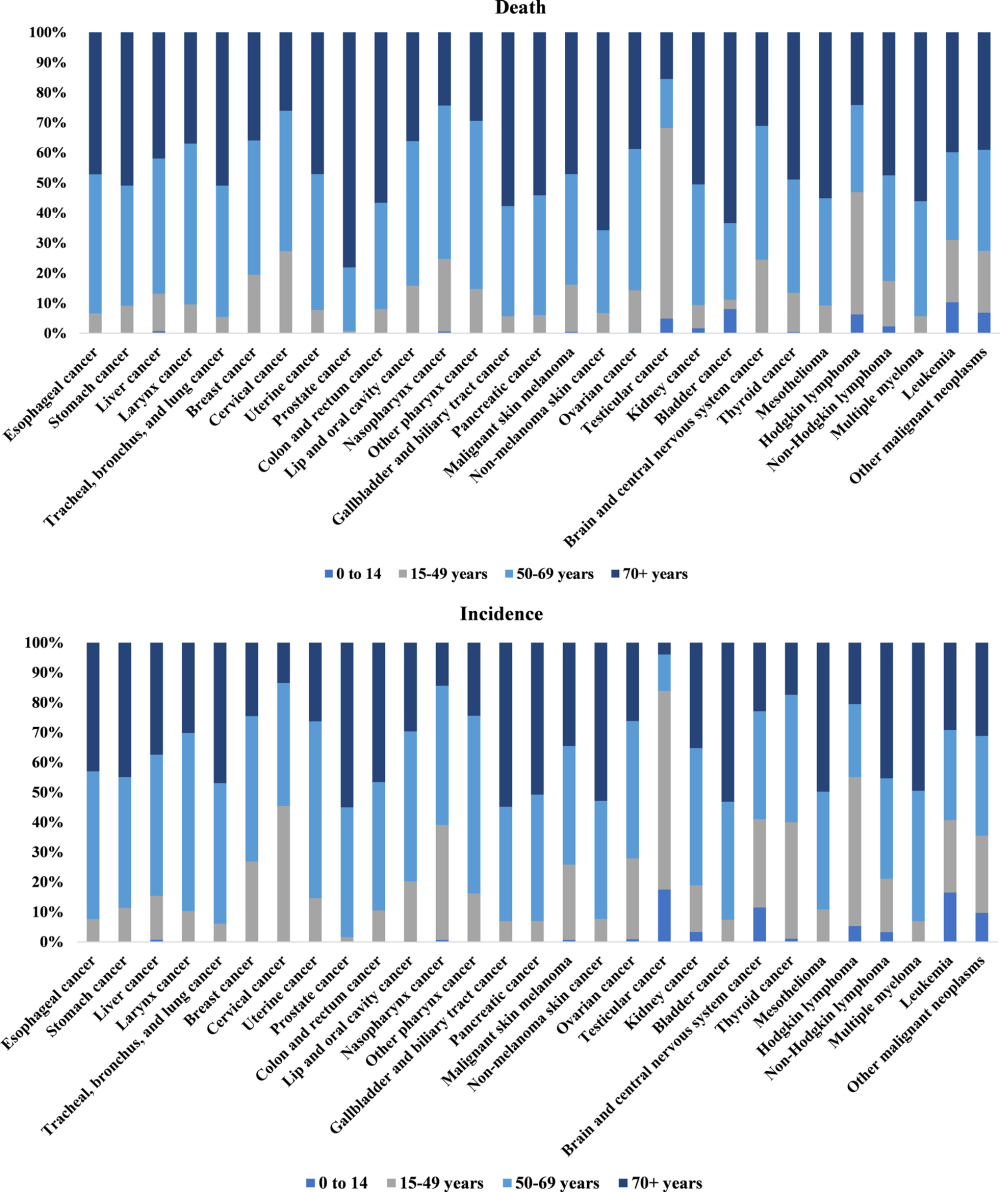
Age-specific global contributions of cancer types to total cancer incidence in 2019

The cancer status of different age groups in 2019 is shown in Fig. 2 and supporting data in Table 2. People over 50 years had the greatest risk of developing cancer, with the number of cases and deaths have accounted for over 85% in all age groups. Among them, 99% of the deaths from prostate cancer occurred in individuals aged over 50. The number of people aged over 50 years who died from esophageal, stomach, larynx, TBL, uterine, colon and rectum, gallbladder and biliary tract, pancreatic, non-melanoma skin, mesothelioma, multiple myeloma and kidney cancers accounted for more than 90% of each cancer total deaths. In addition, up to 63.3% of the population aged 15–49 died from testicular cancer, and 83.8% aged under 50 suffered from the disease. The incidence of Hodgkin lymphoma was also highest in individuals below 50 years, accounting for 55.1% of the total cases. The number of cancer deaths in children (under 15 years of age) was low, accounting for about 1% of the total deaths, and there were fewer types of cancer in children than in adults. However, the proportion of deaths in children under 15 years from leukemia and bladder cancer was 10.3% and 8.0%, respectively. Besides, the incidence of testicular cancer, leukemia, and brain and central nervous system cancer was 17.4%, 16.5%, and 11.5%, respectively, in children under 15.

**Table 2:** The incidence number, ASIR, death number, ASDR and case fatality rate of different regions female breast cancer in 2019

Regions	ASDR	Death number	ASIR	Incidence number	CFR (%)
East Asia	9.12 (11.13–7.36)	98,162 (120,112–79,216)	35.69 (44.54–28.32)	382,321 (477,173–303,308)	25.56
Southeast Asia	19.23 (22.01–16.62)	66,463 (76,424–57,087)	38.52 (44.64–33.11)	138,540 (161,237–118,944)	49.92
Oceania	42.8 (54.23–33.19)	1800 (2288–1377)	65.58 (83.58–50.44)	2990 (3842–2286)	65.26
Central Asia	17.29 (19.16–15.53)	7531 (8410–6746)	38.36 (42.8–34.23)	17,746 (19,917–15,766)	45.08
Central Europe	19.87 (22.71–17.25)	23,042 (26,224–20,121)	60.22 (69.57–52.04)	60,774 (69,893–52,615)	32.99
Eastern Europe	17.47 (20.36–15.05)	34,965 (40,415–30,283)	51.89 (61.31–44.14)	93,968 (110,269–80,449)	33.66
High-income Asia Pacific	9.78 (10.41–8.91)	20,529 (22,336–17,761)	56.3 (67.18–47.14)	97,168 (115,106–80,971)	17.37
Australasia	17.47 (18.69–16.11)	4449 (4799–4005)	84.69 (104.99–68.25)	19,150 (23,737–15,494)	20.62
Western Europe	19.79 (20.77–18.32)	97,509 (103,327–87,377)	85.85 (98.85–74.12)	338,607 (386,967–292,300)	23.05
Southern Latin America	24.04 (25.61–22.41)	11,145 (11,914–10,309)	56.51 (71.94–43.78)	24,595 (31,147–19,218)	42.55
High-income North America	18.36 (19.19–17.28)	60,924 (64,168–56,301)	93.75 (112.64–78.03)	280,020 (334,692–233,430)	19.58
Caribbean	20.84 (24.4–17.62)	5705 (6668–4847)	55.37 (65.11–46.63)	14,940 (17,581–12,616)	37.63
Andean Latin America	12.67 (15.51–10.44)	3762 (4614–3098)	29.63 (36.45–24.05)	8966 (11,032–7271)	42.78
Central Latin America	12.87 (15.09–11.05)	16,681 (19,573–14,310)	38.45 (45.64–32.3)	50,560 (60,048–42,504)	33.48
Tropical Latin America	15.19 (16.11–14.15)	20,299 (21,540–18,912)	39.75 (42.24–37.24)	53,196 (56,529–49,824)	38.21
North Africa and Middle East	15.22 (17.35–13.31)	35,405 (40,571–30,676)	37.48 (42.94–32.68)	94,746 (108,875–82,334)	40.61
South Asia	16.83 (20–13.91)	125,312 (149,357–103,075)	27.72 (33–22.91)	215,790 (256,860–178,051)	60.69
Central Sub-Saharan Africa	22.42 (29.76–16.16)	6846 (9007–4964)	28.98 (38.55–20.86)	9708 (12,820–6954)	77.37
Eastern Sub-Saharan Africa	18.15 (20.6–15.65)	16,395 (18,922–14,024)	24.04 (27.49–20.78)	23,906 (27,839–20,170)	75.5
Southern Sub-Saharan Africa	22.06 (24.54–19.72)	7123 (7946–6329)	33.89 (38.02–30.14)	11,543 (12,971–10,256)	65.08
Western Sub-Saharan Africa	23.25 (28.62–18.65)	24,513 (30,840–19,227)	32.91 (40.11–25.93)	37,976 (46,862–29,485)	70.63
High HDI	17.16 (17.95–16)	205,992 (217,433–186,971)	78.7 (87.05–70.44)	800,199 (884,095–712,146)	21.81
Upper Middle HDI	12.08 (13.47–10.86)	215,208 (240,207–193,510)	39.16 (44.98–34.19)	695,660 (798,468–607,551)	30.86
Lower Middle HDI	18.13 (20.3–15.9)	234,614 (263,527–206,213)	31.71 (35.32–28.16)	431,691 (481,139–383,142)	57.15
Low HDI	18.17 (21.58–15.37)	32,242 (38,568–27,036)	25.31 (30.38–21.11)	48,405 (58,453–40,084)	71.77
High SDI	16.71 (17.45–15.56)	165,968 (175,159–150,337)	79.22 (87.7–70.83)	673,148 (747,674–601,265)	21.09
High-middle SDI	14.93 (16.19–13.75)	163,520 (177,185–150,455)	48.93 (54.49–43.84)	510,299 (567,966–458,379)	30.52
Middle SDI	13.66 (15.18–12.3)	181,116 (201,671–162,719)	35.52 (39.81–31.47)	485,834 (545,187–430,215)	38.46
Low-middle SDI	16.86 (19.24–14.59)	124,911 (142,594–107,972)	29.47 (33.2–25.91)	227,241 (256,008–199,107)	57.2
Low SDI	18.34 (20.84–15.98)	52,546 (60,015–45,728)	25.67 (29.1–22.54)	79,445 (90,893–69,198)	71.46

### Cancer burden in different regions

**Fig. 3:**
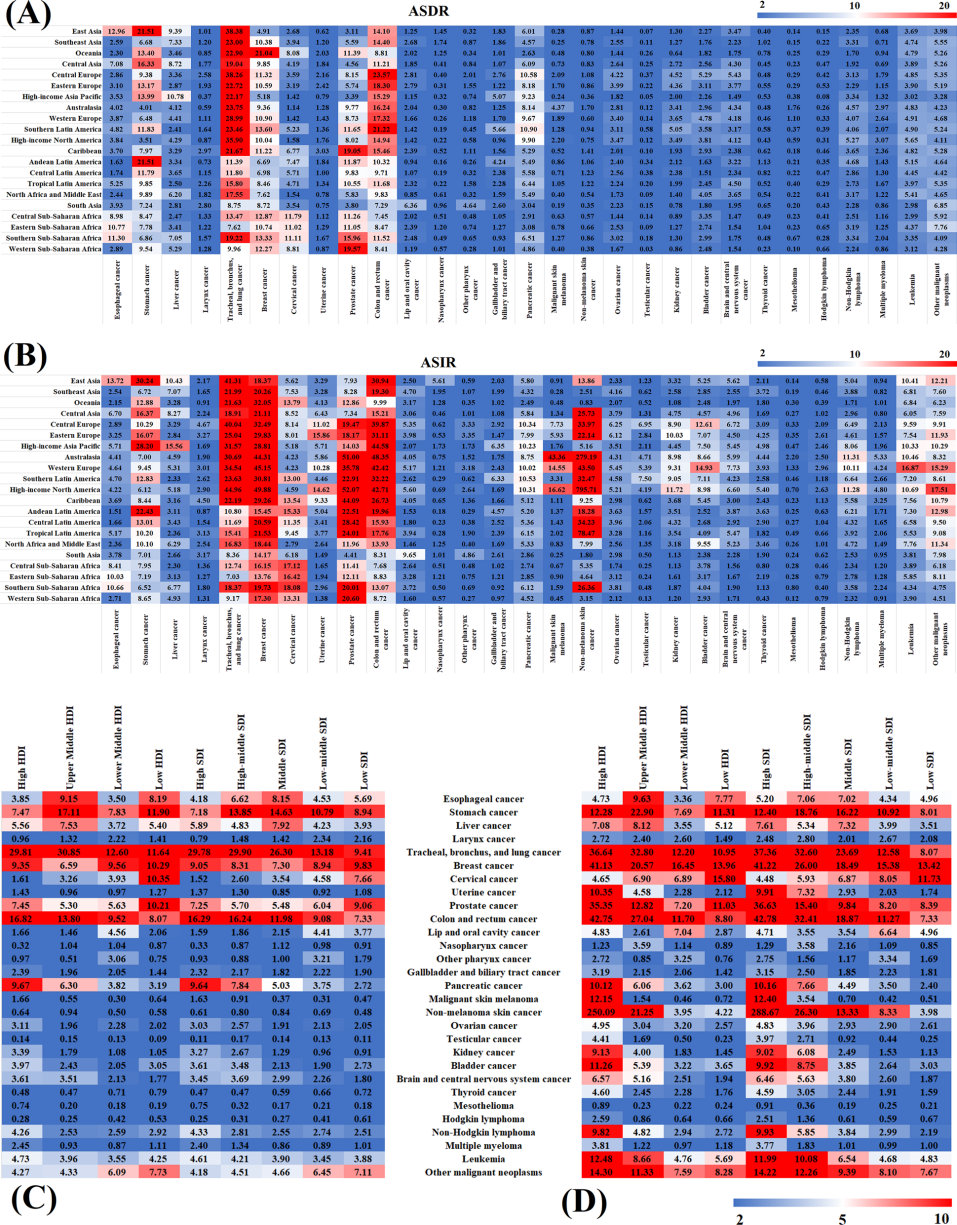
Cancers ranked by age-standardized deaths (**a**) and incidence (**b**) in 21 regions in 2019; and the age-standardized deaths (**c**) and incidence (**d**) of 29 specified cancer groups in 2019 by different HDI and SDI regions

The number of deaths, incidences, ASDR, and ASIR of the 29 cancer types in 21 regions in 2019 were analyzed. The specific results are shown in Fig. 3. In 2019, 2.8 million people died from cancer in East Asia, accounting for 28.0% of the total global deaths. In 1990, 1.5 million people died, which accounted for 26.2% of the total global deaths at the time. TBL cancer, stomach cancer, colon and rectum cancer, and esophageal cancer were the cancers with the higher mortality in East Asia with an ASDR of 38.38 (44.57–32.72), 21.51 (24.95–18.23), 14.10 (16.15–12.24), and 12.96 (15.37–10.19), respectively. Similarly, these cancers also had a high ASIR in East Asia, with 38.38 (44.57–32.72), 21.51 (24.95–18.23), 14.10 (16.15–12.24), and 12.96 (15.37–10.19), respectively. Other regions with the highest number of cancer deaths were Western Europe (1.27 million) and South Asia (1.23 million), which accounted for 12.7% and 12.3% of the global total cancer deaths, respectively. The ASDRs of TBL, colon and rectum, breast, and pancreatic cancer in Western Europe were 28.99 (29.97–27.43), 17.32 (18.13–15.83), 10.90 (11.45–10.01), and 9.67 (10.23–8.95), respectively. The incidences of cancer were mainly concentrated in East Asia, Western Europe, and the high-income North America region, collectively accounting for 63.7% of the world's total cancer incidences. Malignant skin melanoma and non-melanoma skin cancer in these three regions accounted for more than 70% of the total incidences.

The morbidity and mortality rate for different types of cancer also varied among the different regions. The ASDR and ASIR of 29 cancers in 21 regions were compared with global average levels (GAL), and the results were shown in Additional file 1: Figs. S1 and S2. The mortality rate for cervical cancer in Central Sub-Saharan Africa, Eastern Sub-Saharan Africa, and Southern Sub-Saharan Africa was over three times that of GAL. In addition, the mortality rate for prostate cancer in the Caribbean and Western Sub-Saharan Africa was more than three times that of GAL. The ASDR of malignant skin melanoma in Australasia was the highest among all regions [4.37 (5.41–2.96)] and was 5.5 times that of GAL. The ASDR of pancreatic cancer in Southern Latin America and Central Europe was 10.90 (11.74–10.01) and 10.58 (11.90–9.29), respectively, significantly higher than GAL.

Moreover, the mortality rate of testicular cancer in Southern Latin America was four times higher than that of GAL, and Mesothelioma in Australasia and Western Europe was three times higher than that of GAL. The ASIR of malignant skin melanoma in Australasia and non-melanoma skin cancer in high-income North America is more than ten times higher than the GAL. Besides, the ASIR of testicular cancer in Australasia, Western Europe, Southern Latin America, and Central Europe was over three times that of the GAL. In Southern and Eastern sub-Saharan Africa, the ASIRs of esophageal cancer were 11.30 (12.77–10.23) and 10.77 (13.48–8.28), respectively, which was significantly higher than that of the GAL. In addition, the ASIRs of liver cancer in the high-income Asia Pacific and East Asia were 15.56 (17.74–13.46) and 10.43 (12.30–8.76), respectively, which was approximately twice that of GAL. Moreover, the ASIR of testicular cancer in Central Europe and Southern Latin America was about five times that of the GAL, and leukemia in Western Europe was 16.87 (19.38–14.68), which was over two times that of the GAL.

A comparison of the ASDR and ASIR of 29 cancers in 21 regions between 2019 and 1990 revealed that the morbidity and mortality rates in the different regions were different from that of the global trends (Additional file 1: Fig. S3). In contrast to 1990, the global ASDR of liver cancer decreased by 33.4% but was more than two times higher in Central Asia, Australasia, and High-income North America in 2019. Globally, esophageal cancer showed a downward trend, except for Western sub-Saharan Africa, where the mortality rate increased by around 30%. In addition, the mortality rate of breast cancer in Australasia, Western Europe, and high-income North America decreased by over 30%. However, in Oceania, Central Sub-Saharan Africa, and Western Sub-Saharan Africa mortality rate of breast cancer increased by more than 30%.

Similarly, the mortality rate of ovarian cancer, testicular cancer, and brain and central nervous system cancer in the Caribbean increased by more than one times. In contrast to 1990, the morbidity of nasopharynx cancer increased by 37.1% globally. Still, it showed an over 20% decrease in Australasia, Southern Latin America, and South Asia. Besides, colon and rectum cancer morbidity increased by more than one time in East Asia and Andean Latin America. Testicular cancer increased by over two times in East Asia, the Caribbean, Central Latin America, North Africa, and the Middle East.

### Cancer burden on social-economic development

From the perspective of different economic development levels, the incidences and deaths associated with cancer and the distribution of different cancer types were correlated with HDI (Fig. 3c, d). In 2019, the number of cancer deaths in the upper-middle-HDI region exceeded 4 million, accounting for 42.8% of the global cancer deaths, while the low-HDI regions accounted for only 3.7% of the global cancer deaths. Similarly, the high-HDI regions demonstrated the highest cancer incidences, accounting for 51.7% of global cancer incidences. In contrast, the lower-middle- and low-HDI regions accounted for 13.3% and 2.0% of the total global cancer incidences, respectively. The ASDR of the low-HDI regions was 10.35 (12.84–8.03), six times higher than that of the high-HDI regions, and three times higher than that of the upper-middle and lower-middle-HDI regions. The ASDR of prostate cancer in the low-HDI regions was 10.21 (12.14–7.59), which was significantly higher than that in other regions. The mortality rate of TBL cancer in high [ASDR of 29.81 (30.96–27.96)] and upper-middle [ASDR of 30.85 (34.70–27.15)] HDI regions was about three times higher than in the lower-middle- and low-HDI regions. The mortality of colon and rectum cancer, pancreatic cancer, brain, and central nervous system cancers were also significantly high in high and upper-middle-HDI regions than in the lower-middle and low-HDI regions. Similarly, the morbidity of colon and rectum, breast, TBL, prostate, malignant skin melanoma, uterine, and pancreatic cancers, which had ASIR values of 42.75 (46.65–38.54), 41.13 (45.46–36.84), 36.64 (39.94–33.12), 35.35 (50.24–30.03), 12.15 (14.88–8.92), 10.35 (11.53–9.26), and 10.12 (11.06–9.06), respectively, in the high-HDI regions was significantly high than in the other regions. However, among the four HDI regions, the ASIR of cervical cancer [15.80 (19.71–11.90)] was highest in the low-HDI region.

In 2019, the number of cancer deaths in the high-, high-middle-, and middle-SDI regions was 2.66 million, 2.54 million, and 2.9 million, respectively, collectively accounting for over 80% of the total global number of cancer deaths. Some cancers, such as the TBL cancer, demonstrated higher mortality in the three regions, recording ASDRs values of 29.90 (32.60–27.22), 29.78 (30.96–27.82), and 26.30 (29.66–23.01) in high-, high-middle-, and middle-SDI regions, respectively. Similarly, colon and rectum cancer, pancreatic cancer, and brain and central nervous system cancer also recorded a significantly high mortality rate in the three regions. However, cervical cancer, lip and oral cancer morbidity, and other malignant neoplasms in the low-middle- and low-SDI regions were higher than that in the high-, high-middle-, and middle-SDI regions. Moreover, the ASIRs of colon and rectum, breast, TBL, prostate, pancreatic, and kidney cancers in the high-SDI region were 42.78 (46.64–38.75), 41.22 (45.65–36.88), 37.36 (40.77–33.86), 36.63 (52.42–30.84), 10.16 (11.11–9.12), and 9.02 (9.95–8.18), respectively, which were significantly higher than in the high-middle-, middle-, low-middle-, and low-SDI regions. The ASIRs of uterine, prostate and colon and rectum cancers in the high-SDI region were five times that of the low-SDI region. The low-SDI region had the highest morbidity rate of cervical cancer in all the regions, with an ASIR of 11.73 (14.54–9.26).

Based on the CFR analysis (Additional file 1: Fig. S4), the improvement of economic levels caused a gradual decrease in CFR. The CFRs of breast cancer, uterine cancer, prostate cancer, nasopharynx cancer, malignant skin melanoma, non-melanoma skin cancer, testicular cancer, thyroid cancer, Hodgkin lymphoma, and other malignant neoplasms were less than 30% in the high-HDI region. Out of the 29 cancer types, 19 types demonstrated a CFR of less than 50% in the high-HDI region. However, in the low-HDI region, 15 of 29 cancer types recorded a CFR more than 90% the CFRs of esophageal cancer, stomach cancer, liver cancer, TBL cancer, gallbladder, and biliary tract cancer, pancreatic cancer, and non-Hodgkin lymphoma exceeded 100%. As the socio-demographic index increased, the CFR gradually decreased except for pancreatic cancer, whose CFR was above 90% in the high-SDI region, and esophageal cancer, liver cancer, TBL cancer, and pancreatic cancer CFR exceeded 90% in the high-middle-SDI region. However, seven, 10, and 18 cancers in the middle-, low-middle-, and low-SDI regions, respectively, had a CFR above 90%. The CFR of these cancers also varied greatly in the different regions. For example, the CFR of prostate cancer and nasopharynx cancer in the low-SDI region is above 100%, but only about 20% in the high-SDI region.

### Cancer burden in different countries and territories

**Fig. 4:**
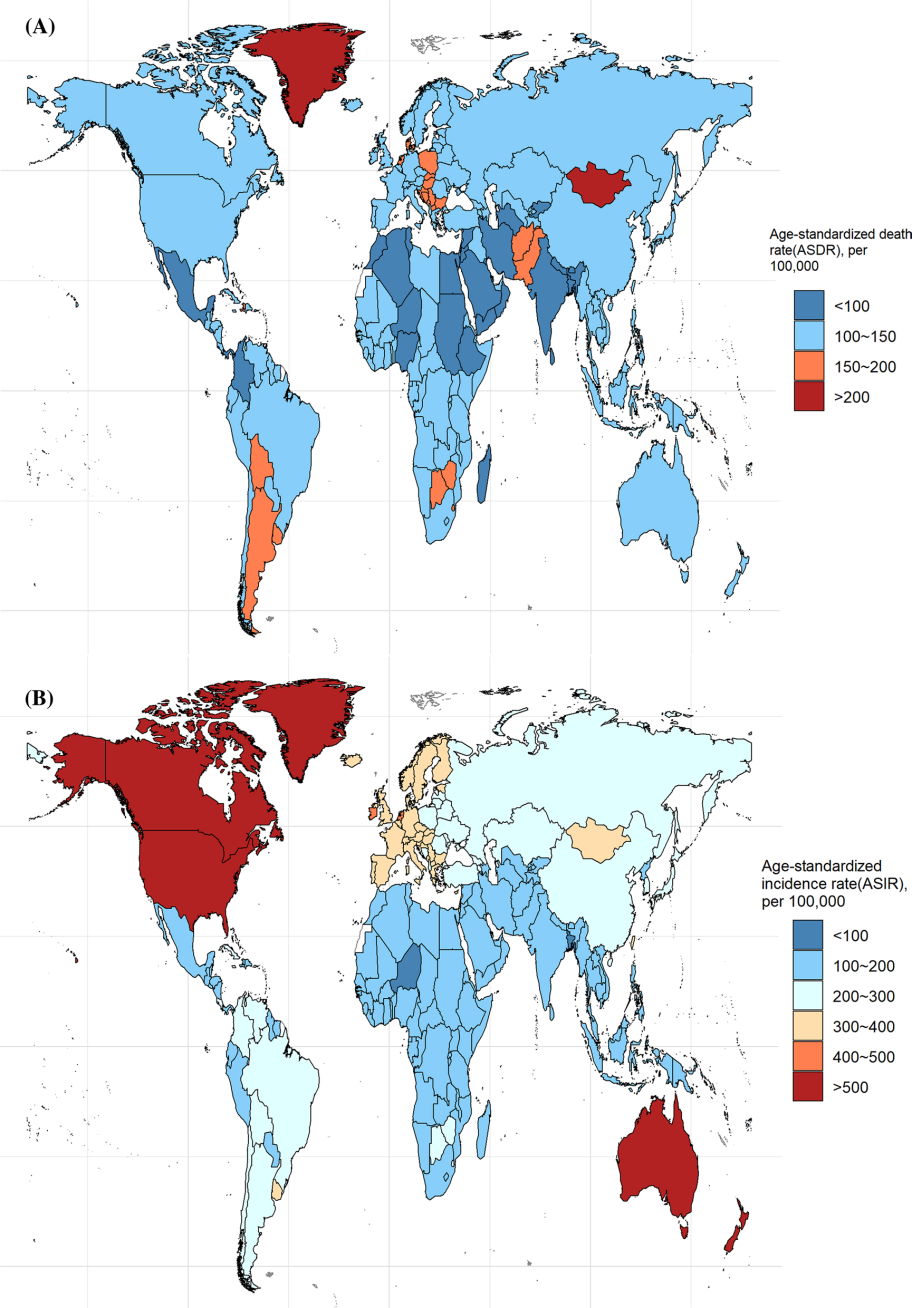
The global disease burden of cancers in 204 countries and territories. **a** ASDR of cancers in 2019; **b** ASIR of cancers in 2019

The ASDRs and ASIRs of the total cancer incidences and deaths in 204 countries and territories in 2019 were statistically analyzed and are presented in Fig. 4. Forty-five countries had ASDRs greater than 150, with the highest being Mongolia, with an ASDR of close to 300 and double that of the GAL. There were 31 countries with ASDRs lower than 100, and Sri Lanka, the Syrian Arab Republic, Algeria, Maldives, Saudi Arabia, and Kuwait showed ASDRs lower than 80. Globally, China had the largest number of cancer deaths (2.71 million), followed by India and the USA, with 930,000 and 760,000, respectively. Besides, 19 countries showed ASIRs greater than 300. Canada and the USA had the highest ASIRs, exceeding 1,000, while Greenland, New Zealand, Australia, and Monaco exceeded 500. Bangladesh and Niger had the lowest morbidity rates, showing ASIRs below 100.

**Fig. 5:**
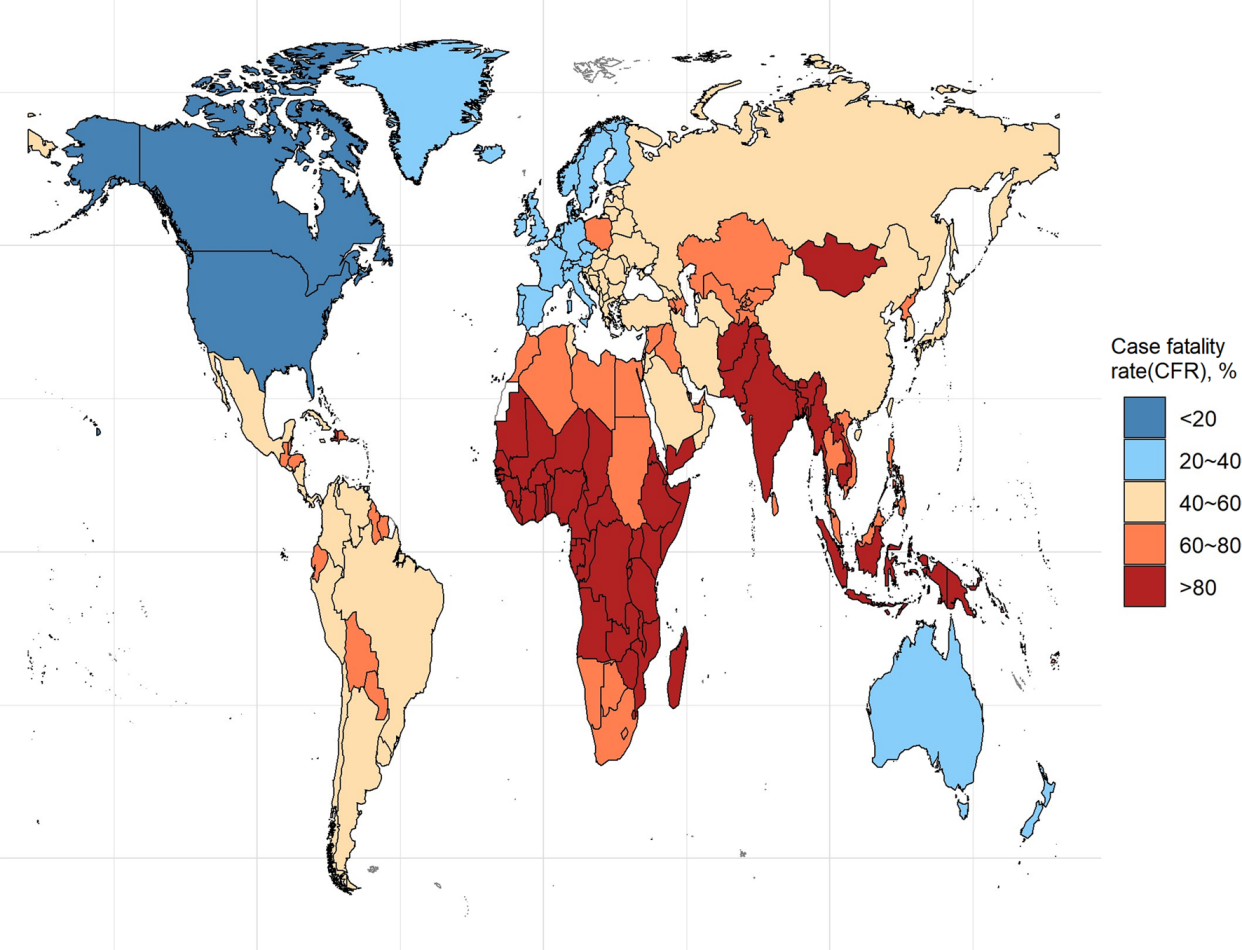
The case fatality rate (CFR) of cancers in 204 countries and territories in 2019

The trends in ASDRs and ASIRs of cancers in 204 countries and territories in 1990 and 2019 were analyzed, and the results are shown in Additional file 1: Fig. S5. In most countries, the ASDRs were declining except for 85 countries where the ASDR increased. Lesotho demonstrated the largest ASDR increase (62.2%), and ASDRS of Cabo Verde, Honduras, Namibia, Kenya, and the Dominican Republic increased by over 30%. The ASDRs of 31 countries decreased by over 20%, with the decrease of Columbia, Czechia, Austria, Kazakhstan, Kyrgyzstan, Bahrain, Bermuda, Luxemburg, and Singapore exceeding 30%. The results showed that the ASIR of most countries and territories (170) had an increasing trend. The largest increase in ASIR was found in Canada (112.8%), and Saudi Arabia, Lebanon, Cabo Verde, Lesotho, Dominic Republic, and Cyprus showed an increase above 50%. The decrease in the ASIRs was relatively small, with only Ethiopia and Kyrgyzstan showing a decrease above 20%. In addition, the CFR analysis of the 29 cancer types revealed that 63 countries and territories had a CFR of more than 80%, among them Chad, Côte d'Ivoire, Kenya, South Sudan, Afghanistan, Central African Republic, Mongolia, Guinea, and Somalia, whose CFRs exceeded 90%. Out of the 240 countries and territories, only 52 recorded a CFR lower than 50%, among them the USA and Canada (10%), and Greenland, New Zealand, and Australia (< 30%) (Fig. 5).

**Fig. 6:**
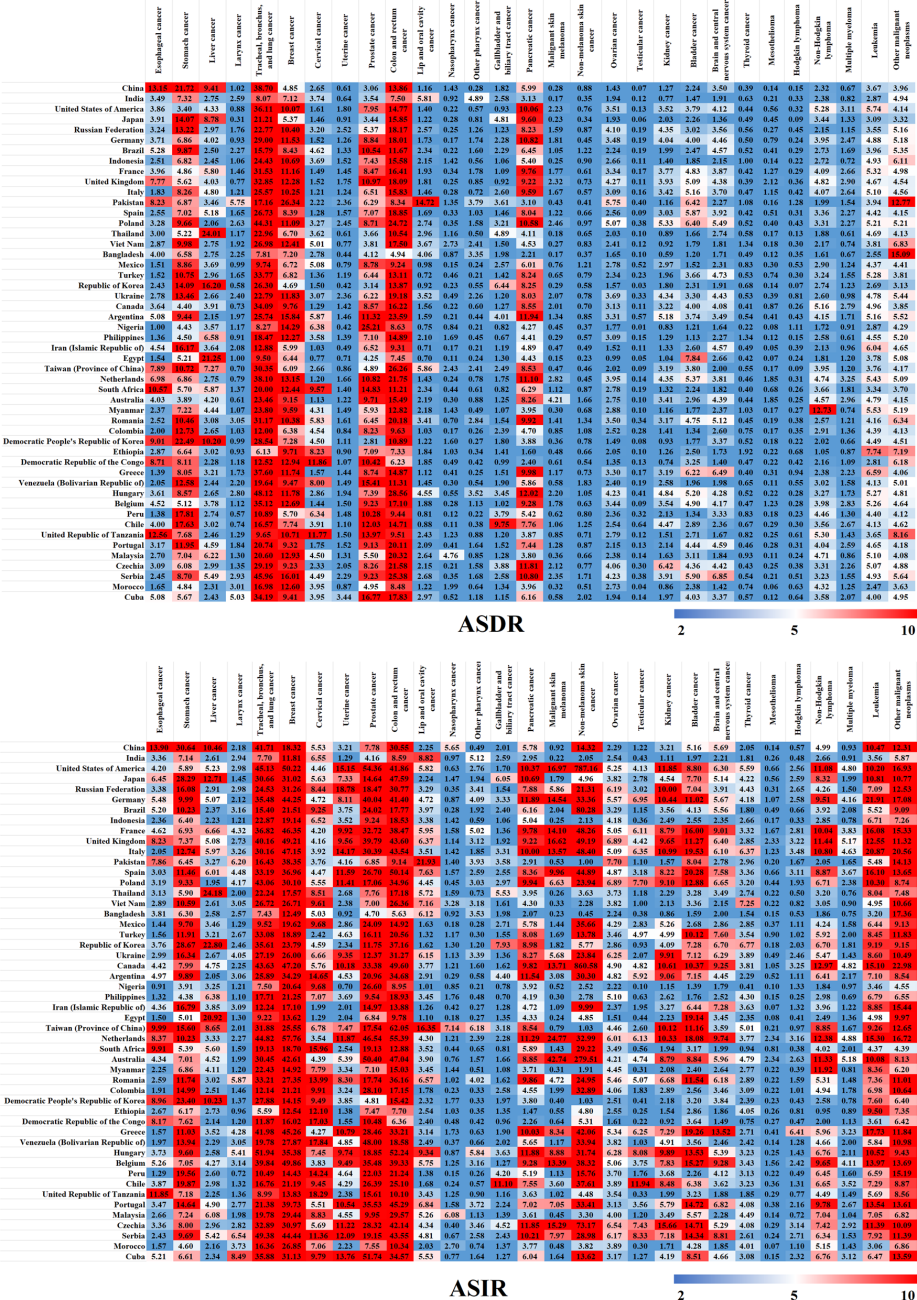
The ASDRs and ASIRs of 29 cancers in 204 countries and territories in 2019 (displays the death numbers for the top 50 countries and territories)

The ASDRs and ASIRs of 29 cancers in 204 countries and territories in 2019 were analyzed, followed by the analysis of the death numbers for the top 50 countries and territories in comparison to the GAL results to check for accuracy (Fig. 6 and Additional file 1: Fig. S6). The mortality rate of stomach cancer, TBL cancer, breast cancer, and colon and rectum cancer was high in all countries and territories. The ASDRs of TBL cancer in Hungary, Serbia, and Poland were 48.12 (58.44–39.58), 45.96 (57.51–36.13), and 44.31 (52.61–36.96), respectively, which were about twice that of GAL. In contrast, the ASDRs of TBL cancer in Bangladesh, Mexico, Nigeria, Egypt, Ethiopia, and the United Republic of Tanzania were lower than 10. In Pakistan, the ASDR of Breast cancer was 26.34 (34.86–20.20), which was more than three times that of the GAL. In addition, Pakistan had higher morbidity of lip and oral cavity, larynx, other pharynx, Hodgkin lymphoma, and testicular cancers, which had ASIRs of 14.72 (18.34–11.90), 5.75 (7.44–4.47), 3.79 (4.91–2.95), 1.28 (1.72–0.94), and 0.40 (0.56–0.29), respectively. The ASIR of lip and oral cavity cancer was six times higher than that of the GAL in Pakistan.

However, the morbidity of liver cancer in most countries was lower than that of GAL, except in Thailand [24.01 (31.65–17.88)], Egypt [21.25 (28.92–15.44)], and Korea [16.20 (17.94–14.47)] whose ASDRs were three to four times that of GAL. Among all cancers, non-melanoma skin cancer had the highest morbidity, especially in Canada [860.58 (1056.62–695.29)] and the USA [787.16 (851.03–723.21)], which were about ten times that of the GAL. Stomach cancer, TBL cancer, breast cancer, and colon and rectum cancer showed the highest morbidity in most countries and territories. For example, the ASIR of breast cancer in the Netherlands and the USA was 57.76 (74.28–44.53) and 50.22 (61.11–41.23), respectively, which was more than twice that of GAL. Besides, the morbidity of stomach cancer was highest in China [30.64 (36.15–25.82)] and Japan [28.29(33.27–23.71)], which was about twice that of the GAL. In 2019, over 600,000 incidences of stomach cancer were reported in China.

### Analysis of significant cancer burden

#### Cancer with the highest CFR—Pancreatic cancer

**Fig. 7:**
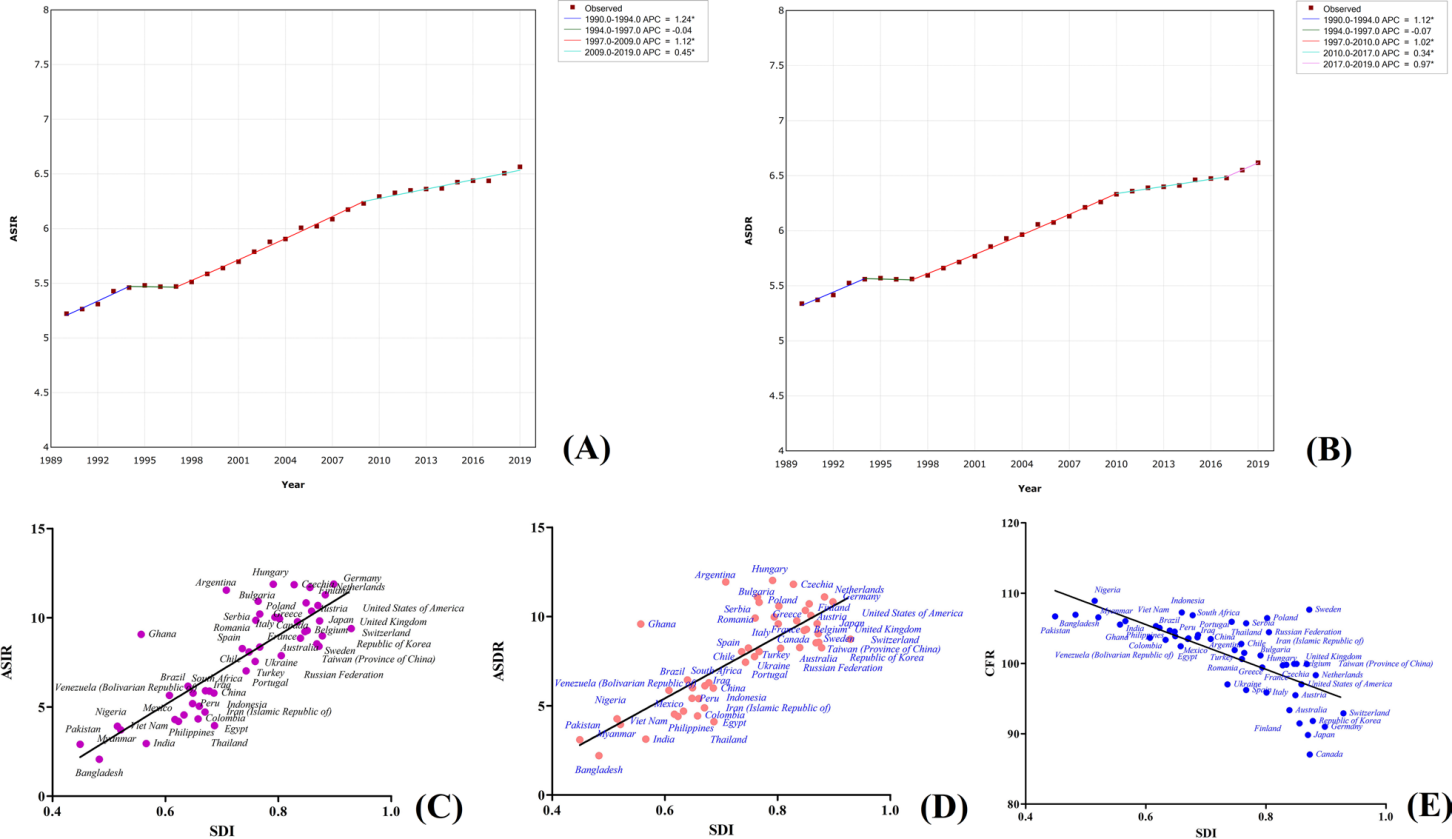
Trends in global morbidity (**a**) and mortality (**b**) of pancreatic cancer from 1990 to 2019; and the correlation of socio-demographic index with morbidity (**c**), mortality (**d**) and CFR (**e**) in different countries and territories

Out of the 29 cancer types, pancreatic cancer had the highest CFR, with an ASDR of 6.62 (7.06–6.11) and 531,107 (566,537–491,948) deaths twice as the number in 1990. In 2019, pancreatic cancer had an ASIR of 6.57 (7.09–6.00) and 530,297 (573,635–486,175) new cases, indicating that its CFR could reach 100%. The pancreatic cancer morbidity and mortality trends globally from 1990 to 2019 are shown in Fig. 7a, b. The ASDR and ASIR of pancreatic cancer showed an upward trend in the past three decades, with an APC of 0.45 between 2009 and 2019 for ASIR, while ASRD had an APC of 0.97 between 2017 and 2019 and 0.34 from 2010 to 2017, implying an increased pancreatic cancer burden between 2017 and 2019. Its morbidity and mortality in males were 1.3 times higher than that of females. The majority of its related deaths (94.0%) and incidences (93.1%) were recorded in people aged over 50. In addition, the mortality of pancreatic cancer was highest in Southern Latin America [10.90 (11.74–10.01)], Central Europe [10.58 (11.90–9.29)], High-income North America [9.90 (10.34–9.27)], Western Europe [9.67 (10.23–8.95)], and High-income Asia Pacific [9.23 (9.85–8.17)], collectively accounting for about 1.5 times that of the GAL. However, the ASDRs for Eastern Sub-Saharan Africa, South Asia, Central Sub-Saharan Africa, and Oceania were 3.08 (3.58–2.65), 3.04 (3.45–2.65), 2.91 (3.59–2.37), and 2.63 (3.22–2.17), respectively. Besides, the ASDRs and ASIRs increased with the increase in the economic index in the different HDI regions. For example, the ASDRs in the high, upper-middle-, lower-middle-, and low-HDI regions were 9.67 (10.18–8.88), 6.30 (6.90–5.68), 3.82 (4.26–3.44), and 3.19 (3.66–2.76), respectively. Similarly, the ASDRs and ASIRs of pancreatic cancer in SDI regions increased with the increase of the socio-demographic index. Countries with the highest morbidity and mortality of pancreatic cancer included the USA, Germany, Argentina, Netherlands, Czechia, Greece, Hungary, Austria, Serbia, Bulgaria, and Finland, with their ASDRs and ASIRs 1.5 times that of GAL. However, the ASDRs and ASIRs for India, Pakistan, and Bangladesh were below half that of the GAL. The SDI in the different countries and territories was positively correlated with the morbidity and mortality of pancreatic cancer but negatively correlated with its CFR based on the death number data in the top 50 countries and territories (Fig. 7c–e and Additional file 1: Table S1).

#### Cancer with the highest mortality—TBL cancer

**Fig. 8:**
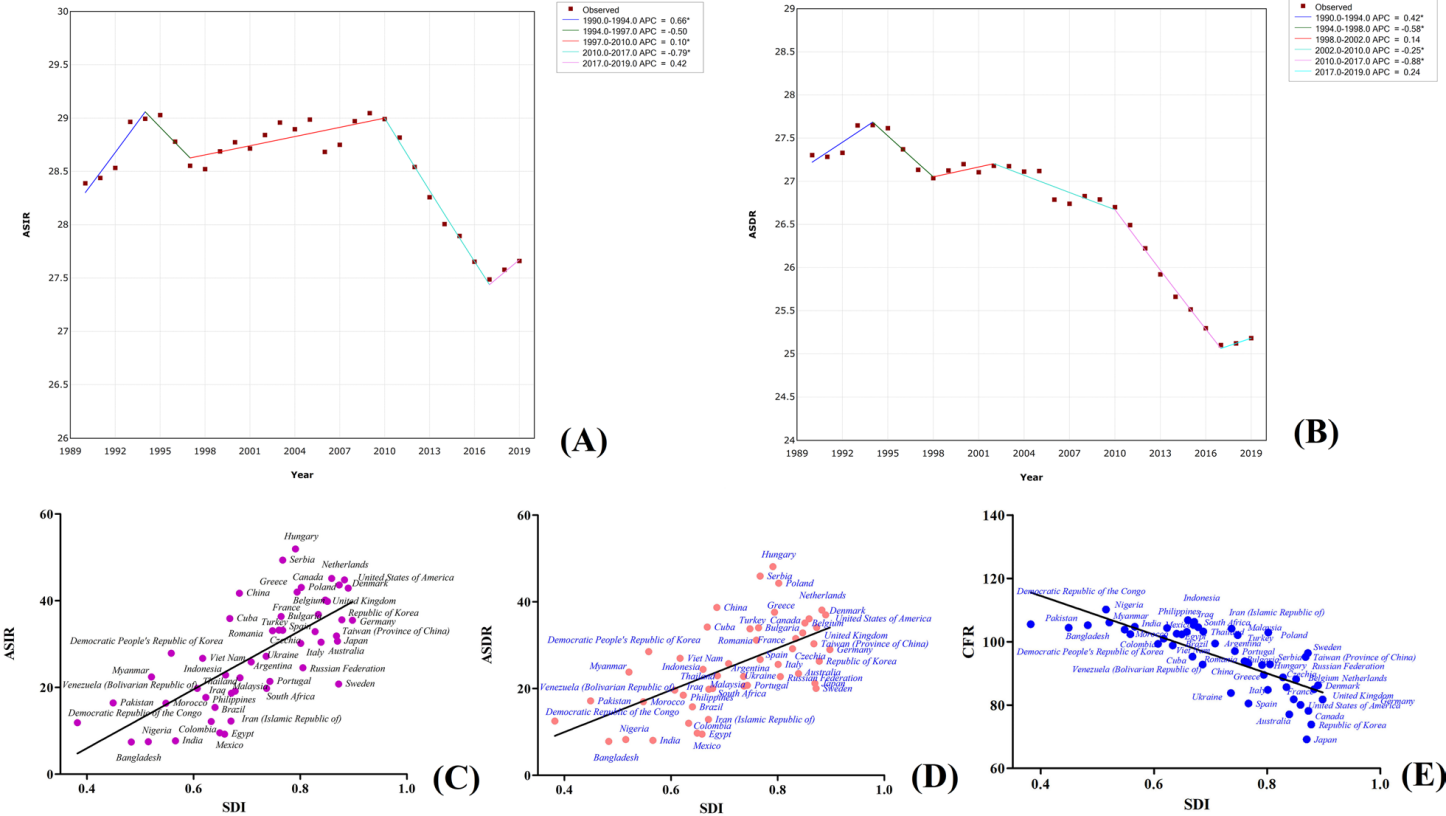
Trends in global morbidity (**a**) and mortality (**b**) of TBL cancer from 1990 to 2019; and the correlation of socio-demographic index with morbidity (**c**), mortality (**d**) and CFR (**e**) in different countries and territories

The mortality rate of TBL cancer is the highest among all cancers types. In 2019, the ASDR of TBL cancer was 25.18 (27.01–23.16), and the number of deaths reached 2,042,640 (2,193,269–1,879,241), which accounted for more than one-fifth of the global cancer deaths. The morbidity and mortality trends of TBL cancer over the past 30 years revealed that the morbidity was consistent from 1990 to 2010, followed by a rapid decrease from 2010 to 2017 (APC = − 0.79). However, the morbidity had an increasing trend from 2017 to 2019, with an APC of 0.42. Similarly, the ASDR recorded an increasing trend from 2017 to 2019 (Fig. 8a, b). The male ASDR due to TBL cancer was 37.38 (40.74–34.09), which was much higher than that of females [14.99 (16.41–13.48)]. Over 90% of the incidences and deaths from TBL cancer were seen in individuals over 50 years. In terms of regions, TBL cancer had the highest mortality rates in East Asia, Central Europe, and High-income North America, recording an ASDR of 38.38 (44.57–32.72), 38.26 (43.52–33.62), and 35.90 (37.29–33.82), respectively. In contrast, its mortality rates in Western Sub-Saharan Africa, South Asia, and Eastern Sub-Saharan Africa were relatively low, with ASDRs of 9.96 (11.62–8.48), 8.75 (10.05–7.44), and 7.62 (9.15–6.45), respectively. The ASDRs and ASIRs in the high- and upper-middle-HDI regions are substantially similar but significantly higher than those in the lower-middle- and low-HDI regions. In addition, the ASDRs and ASIRs in the high- and high-middle-SDI regions were significantly higher than those of the other three regions. Among the countries, China and the USA had the largest number of TBL cancer deaths with 757,171 (887,752–638,741) and 206,196 (214,277–193,717) deaths, respectively), accounting for 50% of the global TBL cancer deaths. Besides, the ASDRs of Poland, Hungary, and Serbia were above 40, with 44.31 (52.61–36.96), 48.12 (58.44–39.58), and 45.96 (57.51–36.13), and were almost twice that of the GAL. However, in India, Mexico, Bangladesh, Nigeria, and Egypt, the morbidity and mortality of TBL cancer are relatively low, and the ASDRs and ASIRs are below 10, which is less than 40% that of the GAL. The correlation between SDI and TBL cancer morbidity, mortality, and CFR in different countries and territories was consistent with that of pancreatic cancer using data from the top 50 countries and territories with the highest death numbers of pancreatic cancer (Fig. 8c–e and Additional file 1: Table S2).

#### The highest cancer burden in females—Breast cancer

**Fig. 9:**
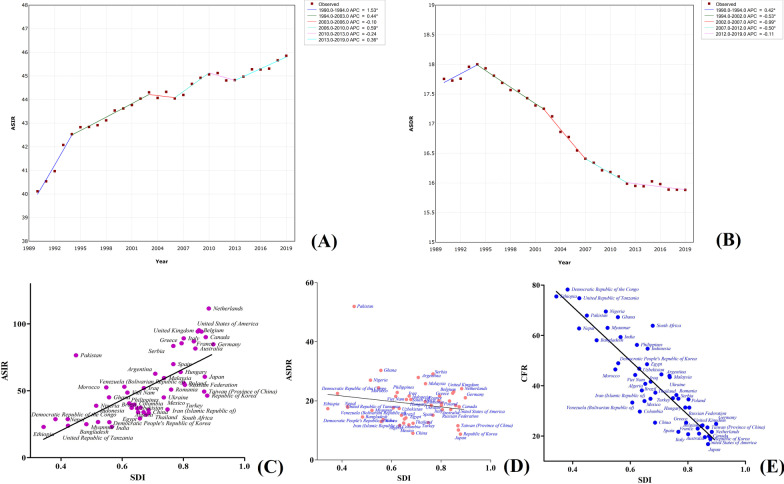
Trends in global morbidity (**a**) and mortality (**b**) of breast cancer in females from 1990 to 2019; and the correlation of socio-demographic index with morbidity (**c**), mortality (**d**) and CFR (**e**) in different countries and territories

Breast cancer is the primary malignant tumor threatening women's health. In 2019, the ASDR and ASIR of breast cancer in women were 15.88 (17.07–14.66) and 45.86 (49.76–41.91), respectively, while the number of incidences and deaths among women reached 1,977,212 (2,145,215–1,807,615) and 688,562 (739,571–635,323), respectively. The morbidity and mortality trend of breast cancer in women from 1990 to 2019 revealed that the morbidity of breast cancer gradually decreased over the years, except for 2003–2006 and 2010–2013. However, mortality showed a downward trend, which gradually flattened from 2012 to 2019 (APC = − 0.11), except from 1990 to 1994 where there was an upward trend (Fig. 9a, b). The majority of the incidences and deaths from breast cancer were mainly in women aged between 50 and 70, accounting for about 50% of the total cases. Among the regions, the largest number of incidences [382,321 (477,173–303,308)] and deaths [98,162 (120,112–79,216)] from breast cancer in women was observed in East Asia, although the region had an ASDR of only 9.12 (11.13–7.36) (Table 2). Oceania had the mortality rate of breast cancer, with ASDR and ASIR of 42.80 (54.23–33.19) and 65.58 (83.58–50.44), respectively. The highest ASIRs of breast cancer were observed in high-income North America [93.75 (112.64–78.03)], Western Europe [85.85 (98.85–74.12), and Australasia [84.69 (104.99–68.25)]. However, the ASDRs and CFR for these regions were low, recording CFRs of 20.6%, 23.1%, and 19.6%, respectively. The highest CFRs of breast cancer in females were observed in African regions, including Central Sub-Saharan Africa (77.37%), Eastern Sub-Saharan Africa (75.50%), Southern Sub-Saharan Africa (65.08%), and Western Sub-Saharan (70.63%). Besides, breast cancer morbidity increased with the increase of economic and socio-demographic indexes in the HDI and SDI regions. However, the mortality rate in these regions was not significantly different, indicating an increase of the economic and socio-demographic indexes, caused a gradual decrease of the CFR of breast cancer. For example, the CFR of high, upper-middle, lower-middle, and low HDI were 21.8%, 30.9%, 57.2%, and 71.8%, respectively. Country-wise, higher female deaths from breast cancer were observed in China [93,499 (115,420–74,511)]. China India, and the USA, collectively accounted for 33.4% of the global breast cancer deaths. However, the highest mortality of breast cancer was observed in Pakistan, whose ASDR was 51.94 (69.76–39.03) and three times that of the GAL. Besides, breast cancer in females had the highest morbidity in the Netherlands, Belgium, USA, UK, and Canada, with ASIRs of 111.49 (143.86–85.92), 95.21 (123.57–73.85), 94.21 (115.12–77.35), 94.29 (118.81–74.32), and 90.10 (115.61–70.12), respectively, which were more than twice that of GAL. Correlation analysis revealed that breast cancer morbidity in the different countries and territories had a strong positive correlation with SDI (Fig. 9c–e). However, CFR and SDI had a strong negative correlation, with the difference between the country and territory with the highest and lowest CFR being about four times based on the data from the top 50 countries and territories with the highest death numbers from female breast cancer (Additional file 1: Table S3).

#### The highest cancer burden in children—Leukemia

**Fig. 10:**
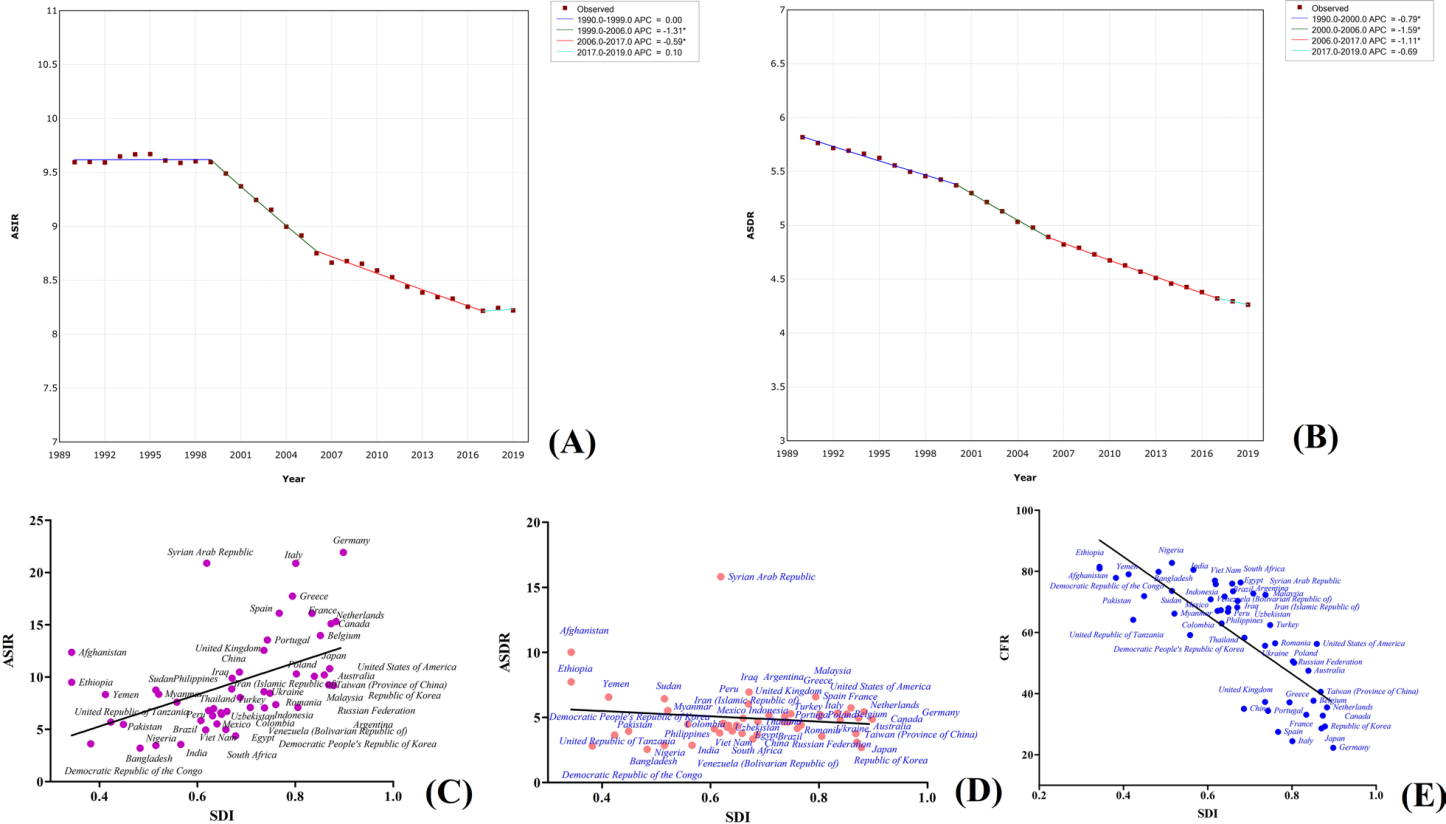
Trends in global morbidity (**a**) and mortality (**b**) of leukemia in females from 1990 to 2019; and the correlation of socio-demographic index with morbidity (**c**), mortality (**d**) and CFR (**e**) in different countries and territories

Leukemia is the main cancer children under 15 years are susceptible to, and its incidences [106,082 (123,151–91,353)] and the number of deaths [34,371 (39,862–29,486)] in this age group accounted for 16.5% and 10.3% of the total leukemia incidences and deaths in all age groups, respectively. In 2019, leukemia caused 334,592 (360,214–306,818) deaths and 643,579 (699,729–586,980) incidences were reported showing ASDRs and ASIRs of 4.26 (4.58–3.91) and 8.22 (8.94–7.5), respectively. Trend analysis from 1990 to 2019 revealed that the morbidity from leukemia remained constant (APC = 0) from 1990–1999, followed by a gradual decrease from 1999–2017, and a slight increase (APC = 0.10) from 2017–2019 (Fig. 10a, b). Overall, mortality from leukemia gradually decreased over the past 30 years. The highest mortalities from leukemia were observed in high-income North America, North Africa and the Middle East, and Andean Latin America, which had ASDRs of 5.65 (5.92–5.28), 5.41 (6.13–4.62), and 5.15 (6.42–3.83), respectively, while low mortality rates were observed in South Asia and Central sub-Saharan Africa, which has ASDRs of 2.98 (3.50–2.59), and 2.99 (4.04–2.18), respectively. The highest morbidity was observed in East Asia, High-income Asia Pacific, Australasia, and High-income North America regions, which had ASIRs greater than 10. However, no significant differences in mortality from leukemia were observed across the different levels of economic development, although the ASDR was slightly higher in high- and low-HDI regions than in other regions. In contrast, the ASIR in the high-HDI region [12.48 (13.79–11.27)] was higher than that of the low-HDI region [5.69 (6.95–4.37)]. A similar trend was also observed in the different SDI regions. Country-wise, the Syrian Arab Republic has the highest ASDR of Leukemia [15.82 (20.41–11.96)], which is more than three times that of the GAL, followed by Afghanistan [10.01 (14.38–6.95)], which is more than twice that of the GAL. In addition, the highest morbidity was observed in Germany, Italy, the Syrian Arab Republic, and Greece, with ASIR more than two times that of GAL. However, the CFR of Germany and Italy was only about 20%, while that in the Syrian Arab Republic was 75.8%. The morbidity and mortality rates of leukemia in India, Nigeria, Bangladesh, and the Democratic Republic of the Congo were significantly lower than that of GAL, but its CFR was above 75%. The SDI in the different countries and territories was positively correlated with leukemia morbidity and negatively correlated with its CFR (Fig. 10c–e). However, leukemia mortality was the same across the different countries and territories based on the data from the top 50 countries and territories with the highest death numbers from leukemia (Additional file 1: Table S4).

#### The most geographically differentiated cancer—Esophageal cancer

**Fig. 11:**
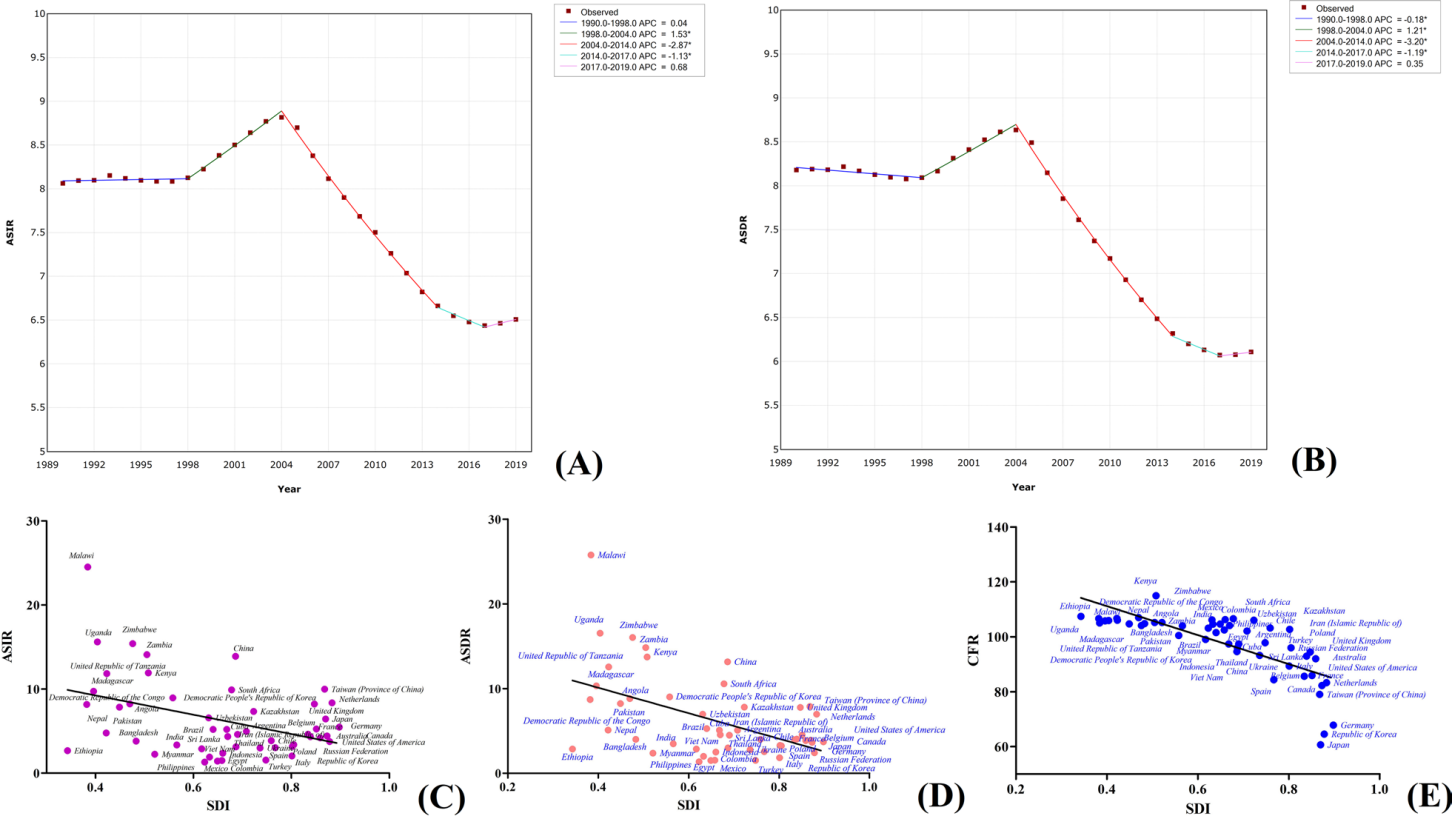
Trends in global morbidity (**a**) and mortality (**b**) of esophageal cancer in females from 1990 to 2019; and the correlation of socio-demographic index with morbidity (**c**), mortality (**d**) and CFR (**e**) in different countries and territories

In 2019, the incidences and deaths from esophageal cancer worldwide reached 500,000, with ASIR and ASDR of 6.51 (7.25–5.69) and 6.11 (6.76–5.38), respectively. The CFR of esophageal cancer (93.9%) comes second after that of pancreatic cancer. Trend analysis of esophageal cancer morbidity and mortality over the past 30 years revealed that there were no significant differences from 1990 to 1998, followed by a significant increase from 1998 to 2004, rapid decline from 2004 to 2017, and finally a gradual increase from 2017 to 2019 (Fig. 11a, b). Regionally, the highest number of deaths from esophageal cancer was observed in East Asia [263307 (314,860–209,014)], which accounted for 53% of the global total esophageal cancer deaths in 2019. East Asia had an ASDR of 12.96(15.37–10.19), which was about eight times that of Andean Latin America [1.63(2.00–1.33)]. Other regions with a higher mortality rate from esophageal cancer were Southern and Eastern Sub-Saharan Africa, with an ASDR of 11.30 (12.77–10.23) and 10.77 (13.48–8.28), respectively. Similarly, their morbidity was significantly higher than in the rest of the regions, with ASIR of 13.72 (16.25–10.64), 10.66 (12.29–9.56), and 10.03 (12.60–7.71), respectively. Compared to the other cancer types, esophageal cancer morbidity and mortality patterns in the HDI and SDI regions were different. For example, the ASDR in the upper-middle-, low-, and high-HDI regions was 9.15 (10.66–7.41), 8.19 (9.87–6.20), and 3.85 (4.02–3.63), respectively. Among the countries, esophageal cancer caused the largest number of deaths [257,316 (309,029–202,777)] and incidences [278,121(331,600–213,512)] in China, which accounted for more than 50% of the total esophageal cancer deaths globally. Besides, high mortality and morbidity of esophageal cancer were observed in China, with ASDR and ASIR of 13.15 (15.68–10.27) and 13.90 (16.52–10.70), respectively, which were more than twice that of GAL. Other countries where a high mortality rate was observed include the United Republic of Tanzania, Kenya, Uganda, Malawi, Zimbabwe, and Zambia, with ASDR more than twice that of GAL. Among these countries, Malawi had the highest ASDR [25.76 (33.94–19.76)]. Correlation analysis revealed that the SDI of different countries and territories were negatively correlated with esophageal cancer morbidity, mortality, and CFR (Fig. 11c–e and Additional file 1: Table S5). The differences in morbidity and mortality of esophageal cancer in different sexes were also significant, where males had an ASDR of 9.68 (10.96–8.34) and 3.02 (3.43–2.52) in females.

## Discussion

In this study, we assessed the global burden of 29 cancers in 2019. The analysis of the morbidity, mortality, incidences, and deaths by region, country, and gender revealed that the cancer spectrum varies widely among the different regions and countries. In addition, some cancer types were analyzed individually to quantify and clarify the burden of the specific cancers. Although the global cancer deaths exceeded 10 million in 2019, nearly onefold of the deaths in 1990, the mortality rate of cancer decreased by 15% due to the rapid global population growth. However, the global cancer incidences in 2019 were 2.3 times the number observed in 1990, an 11% increase compared to 1990. Thus, the increased morbidity of cancer observed in 2019 implies a need for better global means of cancer prevention under the current aggravating risk factors. However, the observed reduction of cancer mortality implies that the nearly 30 years of medical development from 1990 has achieved good results in cancer treatment, for example, in the treatment of stomach cancer and Hodgkin lymphoma, whose mortality declined by over 40%. The decrease in mortality from stomach cancer is mainly related to stomach cancer screening and treatment methods. For example, gastric cancer detected in the early stages can be treated radically under endoscopy, exceeding the 5-year survival rate by over 90%. In addition, the morbidity of stomach cancer had the largest decline of over 30% among all the cancers, mainly due to the decrease in the infection rate of *Helicobacter pylori*, salt intake, and increased intake of fresh vegetables and fruits [11, 12].

The significant differences in the cancer spectrum among regions can be attributed to the local environment, living habits, medical conditions, etc. For example, in resource-rich and developed Western countries, the morbidity and mortality of cervical cancer have slowly declined, where over 80% of cervical cancer deaths are reduced through prevention strategies and screening [13]. However, in Africa, the morbidity and mortality rates of cervical cancer are relatively high due to the lack of established scientific and effective screening schemes for cervical cancer, low vaccination rates, the prevalence of AIDS, and limited medical resources [14, 15].

Among the cancer types, pancreatic cancer had the highest CFR; this is because pancreatic cancer is highly invasive, highly malignant, has a low resection rate, and has a very poor prognosis [16]. Besides, it lacks specific tumor markers, making it difficult to diagnose early using the current imaging technologies, which are also unsuitable for large-scale screening. Pancreatic cancer is common in people over 50 years. Its incidences are increased by risk factors such as smoking, chronic pancreatitis, obesity, and diabetes [17]. The pancreatic cancer risk ratio is 2.5 times more in smokers, and quitting smoking for two years reduces this risk by 48%. However, smokers must quit smoking for at least ten years for pancreatic cancer risk to drop to the level of non-smokers [18]. Besides, type 2 diabetes has been linked to an increased risk of pancreatic cancer, where 9.7% of pancreatic cancer occurrences in Italy have been attributed to diabetes [19]. A history of diabetes for five years or more increases the relative risk for pancreatic cancer by 2.1 times [20], with 8.8% of pancreatic cancer deaths been related to a high fasting plasma glucose [21]. In addition, a high BMI index and exposure to chloride, metals and textile dust, and organic solvents increase the risk of pancreatic cancer [22]. In addition, various types of chronic pancreatitis, including alcoholic, non-alcoholic, hereditary, and tropical, have been associated with pancreatic cancer incidences [23, 24]. Surgical resection is the only effective way to cure and give a chance of long-term survival in pancreatic cancer patients. However, more than 80% of pancreatic cancer patients miss surgery since the cancer is discovered at the terminal stages. Therefore, prevention strategies are fundamental to reduce the pancreatic cancer burden. In addition, future strategies, including comprehensive policies to control tobacco use, alcohol intake, and measures to reduce obesity and diabetes burden across the world, should be enacted. More importantly, patients with pancreatic duct stones, intraductal mucinous papillomas, cystic adenoma, and other benign pancreatic lesions should seek medical attention earliest possible.

Globally, the incidences of female breast cancer in most regions such as South America and Africa are still rising, reflecting the impact of social-economic development on female breast cancer [25]. The risk factors of breast cancer are related to non-breastfeeding, short periods of breastfeeding, early age at menarche, late menopause, nulliparity, obesity, alcohol consumption, smoking, long-term exposure to exogenous estrogen, and high intake of red meat, animal fats, and refined carbohydrates [26–29]. For example, the risk of breast cancer in age at menarche below 12 is about twice higher than in over 12, while women with first childbirth age over 30 have more than six times the risk of breast cancer than those less than 30 [29, 30]. Although childbirth reduces the risk of breast cancer, this risk is increased by 80% five years after childbirth compared to nulliparous. However, this association crossed over from positive to negative about 24 years after birth [31]. Besides, a Western-like diet rich in calories increases breast cancer risk by about 14% [32]. However, although breast cancer incidences in these regions are high, the mortality rate is low due to the sufficient local medical resources and effective prevention and control practices. The breast cancer prevention strategy entails strengthening the primary prevention practices, such as abstaining from alcohol and tobacco consumption, balanced and healthy nutrition, maintaining healthy body weight, exercising, timely childbirth, and breastfeeding.

TBL cancer was the most common and leading cause of death among all cancer types. The morbidity and mortality of TBL cancer were significantly different between males and females. The global morbidity of TBL cancer in males was 2.4 times higher than that of females, mainly due to the smoking habit in males. In addition, the number of deaths from TBL cancer was over 90% in countries with higher smoking rates [33]. Passive smoking and environmental pollution, including indoor pollution, also significantly contribute to the TBL cancer burden [34]. In addition, outdoor air pollution, such as high levels of fine particulate matter (PM2.5), is the leading cause of the high TBL cancer burden in China [35]. However, the morbidity and mortality of TBL cancer in most developed countries showed a stable decline since these countries adopted tobacco control measures relatively early [36]. In China, the morbidity of TBL cancer is high, with significant gender differences due to a large number of male smokers compared to females. The incidence of TBL cancer in Chinese women is related to exposure to indoor air pollution from coal heating and cooking fumes [37, 38]. Therefore, there is a need to develop prevention and control strategies for indoor and outdoor particulate pollution. In addition, glucose levels also need to pay more attention to control, because the fasting glucose levels and diabetes patients have a higher incidence and death from TBL cancer [39].

Leukemia is the leading cause of cancer-related deaths in children under 15 years, accounting for about 34.8% of deaths from malignant tumors in children. The incidences of leukemia in industrialized countries are significantly higher than in non-industrialized countries, implying leukemia is closely related to environmental exposure [40, 41]. However, the etiology of childhood leukemia is still unknown, but it is believed to be related to the combined effect of the physical, chemical, biological, and other factors. The risk factors for leukemia include maternal/paternal smoking, maternal leukemia, and exposure to alcohol, tobacco, and pesticides during pregnancy [42, 43]. A meta-analysis revealed that current and ever smokers have a higher risk of acute myeloid leukemia than non-smokers [44]. Besides, the chemicals in tobacco smoke increase the chances of micronuclei formation and chromatid exchange in myeloid tissues [45]. Nevertheless, smoking is positively associated with shorter remission, survival, and lung infection during leukemia treatment [46]. Indoor use of pesticides during the first three months of pregnancy and in the first three years of a child, in addition to extremely low-frequency electromagnetic radiation and exposure to polycyclic aromatic hydrocarbons (PAHs) indoors, increases the risk of acute childhood leukemia [47–49].

Benzene and formaldehyde are recognized as leukemia risk factors that can induce leukemia-related cytogenetic changes in myeloid progenitor cells [40, 50]. Thus, leukemia prevention measures include avoiding environmental contact with benzene and related chemicals, avoiding or reducing contact with ionizing radiations such as X-ray and γ-ray, and abstaining from smoking. However, future research should focus on the etiology of childhood leukemia to effectively identify the risk factors to enhance the prevention and control of leukemia in children.

Esophageal cancer also had a high CFR but with significant regional differences. The "Asian esophageal cancer belt" (eastern Turkey, Caspian coastal countries, northern Afghanistan, Eastern and Central Asia), Japan, South Africa, and South America have high incidences of esophageal cancer [51]. For example, the number of esophageal cancer deaths and incidences in China accounted for over 50% of the global esophageal cancer cases. However, the morbidity and mortality of esophageal cancer greatly vary across China. They are lower in urban areas than in rural areas. In addition, the morbidity and mortality rates of esophageal cancer were highest in the central and the lowest in the east of China [52]. Drinking tea at high temperatures and excessive alcoholism or smoking is the leading cause of high morbidity of esophageal cancer in China [53]. The risk factors of esophageal cancer in China include smoking, alcohol uptake, low intake of vegetables and fruits. Besides, in African countries such as Western sub-Saharan Africa, characterized by higher incidences of esophageal cancer, they have a low supply of Fe, Mg, Zn, and Se in their diets [54, 55]. In women, a high body mass index (BMI) and a low-fruit diet are the main risk factors in China [56]. Moreover, a high BMI is an established risk factor for esophageal adenocarcinoma [57]. Barrett esophagus (BE) is a precancerous lesion of esophageal adenocarcinoma, with BE patients having 30–40 times increased risk of esophageal adenocarcinoma than the general population. For example, 86% of patients with esophageal adenocarcinoma have a history of BE; thus, the incidence rate of esophageal adenocarcinoma is related to BE incidence, which explains the high incidence of esophageal cancer in Asia [58–60]. Therefore, there is a need for cytological and endoscopic screening for early detection of esophageal cancer and precancerous lesions among the high burden countries, such as China [61]. Besides, the primary prevention measures against the risk factors should be strengthened.

There are some differences between the data estimated from GBD and GLOBOCAN. For example, according to GBD, there were 534,364 new cases of liver cancer and 484,577 deaths from liver cancer in 2019. However, based on GLOBOCAN estimates, there were 905,677 and 830,180 new and death cases in 2020, respectively. Similarly, GBD estimates of incidence and death from stomach cancer were 1,269,806 and 957,185 in 2019, respectively, and 1,089,103 and 768,793, respectively, in 2020 GLOBOCAN estimates. Leukemia and Thyroid cancer incidence estimates from GBD were 643,579 and 233,847, respectively, in 2019, and 474,519 and 586,202, respectively, in 2020 based on GLOBOCAN. Moreover, there were significant differences in the different databases for same cancer and country. For example, the pancreatic cancer morbidity estimates from the GBD were 2.95, 6.12, 6.29, 3.10, and 4.27 in India, Iraq, South Africa, Pakistan, and Nigeria in 2019, and 0.94, 2.9, 4.2, 0.73, and 1.6, respectively, in 2020 based on the GLOBOCAN estimates. In addition, the breast cancer morbidity from the GBD were 32.08, 29.31, 37.27, and 39.64 in South Africa, Egypt, Uzbekistan, and Brazil in 2019, and 52.6, 48.7, 26.4, and 61.9, respectively, in 2020 based on the GLOBOCAN estimates [1]. These differences can be attributed to the different estimation methods of cancer incidence and mortality in the two databases. With GLOBOCAN, the cancer incidence is estimated first, followed by survival modeling per country to estimate mortality. On the contrary, GBD uses cancer registry incidence-based mortality estimates and vital registration data to model mortality. The mortality estimates and modeled mortality to incidence ratio (MIR) are then used to estimate the cancer incidence. Thus, a GBD study is advantageous because it allows for trend comparisons over time, allowing for analyzing the demographical and epidemiological transition. Besides, countries with few high-quality population-based cancer registries also lead to discrepancies between GLOBOCAN and GBD estimates [62, 63]. Some researchers have also highlighted that GLOBOCAN and GBD estimates are inaccurate in some countries and regions [64, 65]. Therefore, to improve data accuracy, establishing of local infrastructure to develop high-quality population-based cancer registries in low-income and middle-income countries should be promoted.

## Conclusion

Cancer is expected to become the leading cause of death in every country in the twenty-first century. The incidences of cancer greatly vary in the different regions and countries around the world. These differences are a factor of population composition, society, economy, and lifestyle in the different regions and countries. Besides, the risk factors for specific cancer types were also variant in the different regions and countries. Therefore, when formulating cancer prevention and treatment strategies, more focus should be on the local characteristics and prevailing risk factors. In addition, more emphasis should be put on the importance of cancer screening and the quality of cancer treatment improved to narrow the gap in cancer burden between the different regions and countries, gradually and steadily reducing the global cancer burden. At the same time, cancer prevention and health education should be strengthened, increase public awareness of cancer risk factors, and conduct targeted screening of the different cancer types to reduce the risk of cancer and relieve the cancer burden.

## Limitations

Although the GBD estimates fill the gap for the inaccessibility of actual data on the cancer burden, some limitations are still noted. First, the accuracy of GBD estimation depends largely on the quality and quantity of data used since a low proportion of the population is covered during cancer registrations in some regions such as South America, Asia, and Africa, resulting in great uncertainty in these estimates. Secondly, the increasing trend of cancer burden is also related to the significant increase of cancer registries compared to the past, leading to misinterpretation of such trends. Lastly, there are under-reporting and under-diagnosis during cancer registration, especially in less developed countries, leading to underestimating cancer incidences and deaths.

## Supplementary Information

**Additional file 1: Fig. S1**. The age-standardized deaths of cancers in 21 regions compared with those globally in 2019. **Fig. S2**. The age-standardized incidence of cancers in 21 regions compared with those globally in 2019. **Fig. S3**. Comparison of the ASDR(A) and ASIR(B) of 29 cancers in 21 regions between 1990 and 2019. **Fig. S4**. The case fatality rate (CFR) of cancers in different HDI and SDI regions. **Fig. S5**. The relative changes in ASDR and ASIR of 29 specified cancer groups between 1990 and 2019. **Fig. S6**. The age-standardized deaths and incidence of cancers in different countries and territories compared with the global in 2019. (Displays the death numbers for the top 50 countries and territories). **Table S1**. Death numbers, incidence numbers, ASDR, ASIR, and GAL of pancreatic cancer of 50 countries and territories in 2019. **Table S2**. Death numbers, incidence numbers, ASDR, ASIR, and GAL of TBL cancer of 50 countries and territories in 2019. **Table S3**. Death numbers, incidence numbers, ASDR, ASIR, and GAL of female breast cancer patients in 50 countries and territories in 2019. **Table S4**. Death numbers, incidence numbers, ASDR, ASIR, CFR, and GAL of leukemia in 50 countries and territories in 2019. **Table S5**. Death numbers, incidence numbers, ASDR, ASIR, and GAL of esophageal cancer of 50 countries and territories in 2019.

## Data Availability

The datasets generated and analyzed during the current study are available at the GBD repository (http://ghdx.healthdata.org/gbd-results-tool).

## Abbreviations

APC: Annual percentage change
ASDR: Age-standardized death rate
ASIR: Age-standardized incidence rate
BE: Barrett esophagus
BMI: Body mass index
CFR: Case fatality rate
CI5: Cancer Incidence in Five Continents
GAL: Global average levels
GBD: Global Burden of Disease
HDI: Human Development Index
ICD: International Classification of Diseases
MIR: Mortality-to-incidence ratios
NCI: National Cancer Institute
PAHs: Polycyclic aromatic hydrocarbons
SDI: Socio-demographic index
TBL: Tracheal, bronchus, and lung
UI: Uncertainty interval
WHO: World Health Organization
