# Supplementary material for: Global, regional, and national cancer incidence and death for 29 cancer groups in 2019 and trends analysis of the global cancer burden, 1990–2019
Source: J Hematol Oncol. 2021 Nov 22;14:197. doi: 10.1186/s13045-021-01213-z (PMC8607714; doi:10.1186/s13045-021-01213-z)
Supplement: Supplementary file 1 — Additional file 1: Fig. S1. The age-standardized deaths of cancers in 21 regions compared with those globally in 2019. Fig. S2. The age-standardized incidence of cancers in 21 regions compared with those globally in 2019. Fig. S3. Comparison of the ASDR(A) and ASIR(B) of 29 cancers in 21 regions between 1990 and 2019. Fig. S4. The case fatality rate (CFR) of cancers in different HDI and SDI regions. Fig. S5. The relative changes in ASDR and ASIR of 29 specified cancer groups between 1990 and 2019. Fig. S6. The age-standardized deaths and incidence of cancers in different countries and territories compared with the global in 2019. (Displays the death numbers for the top 50 countries and territories). Table S1. Death numbers, incidence numbers, ASDR, ASIR, and GAL of pancreatic cancer of 50 countries and territories in 2019. Table S2. Death numbers, incidence numbers, ASDR, ASIR, and GAL of TBL cancer of 50 countries and territories in 2019. Table S3. Death numbers, incidence numbers, ASDR, ASIR, and GAL of female breast cancer patients in 50 countries and territories in 2019. Table S4. Death numbers, incidence numbers, ASDR, ASIR, CFR, and GAL of leukemia in 50 countries and territories in 2019. Table S5. Death numbers, incidence numbers, ASDR, ASIR, and GAL of esophageal cancer of 50 countries and territories in 2019. [file 13045_2021_1213_MOESM1_ESM.docx]

Additional file fig S1. The age-standardized deaths of cancers in 21 regions compared with those globally in 2019.


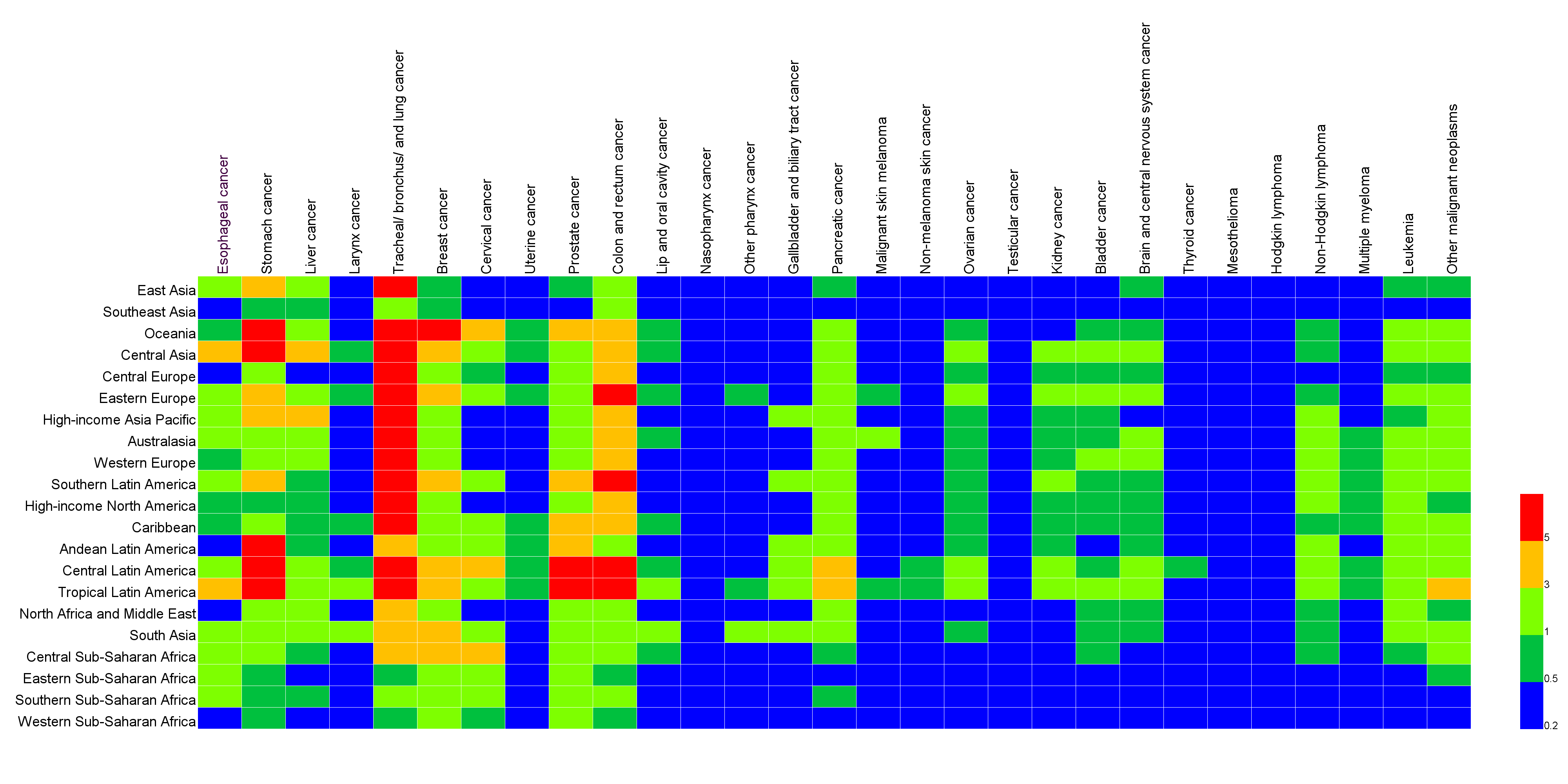


Additional file fig S2. The age-standardized incidence of cancers in 21 regions compared with those globally in 2019.


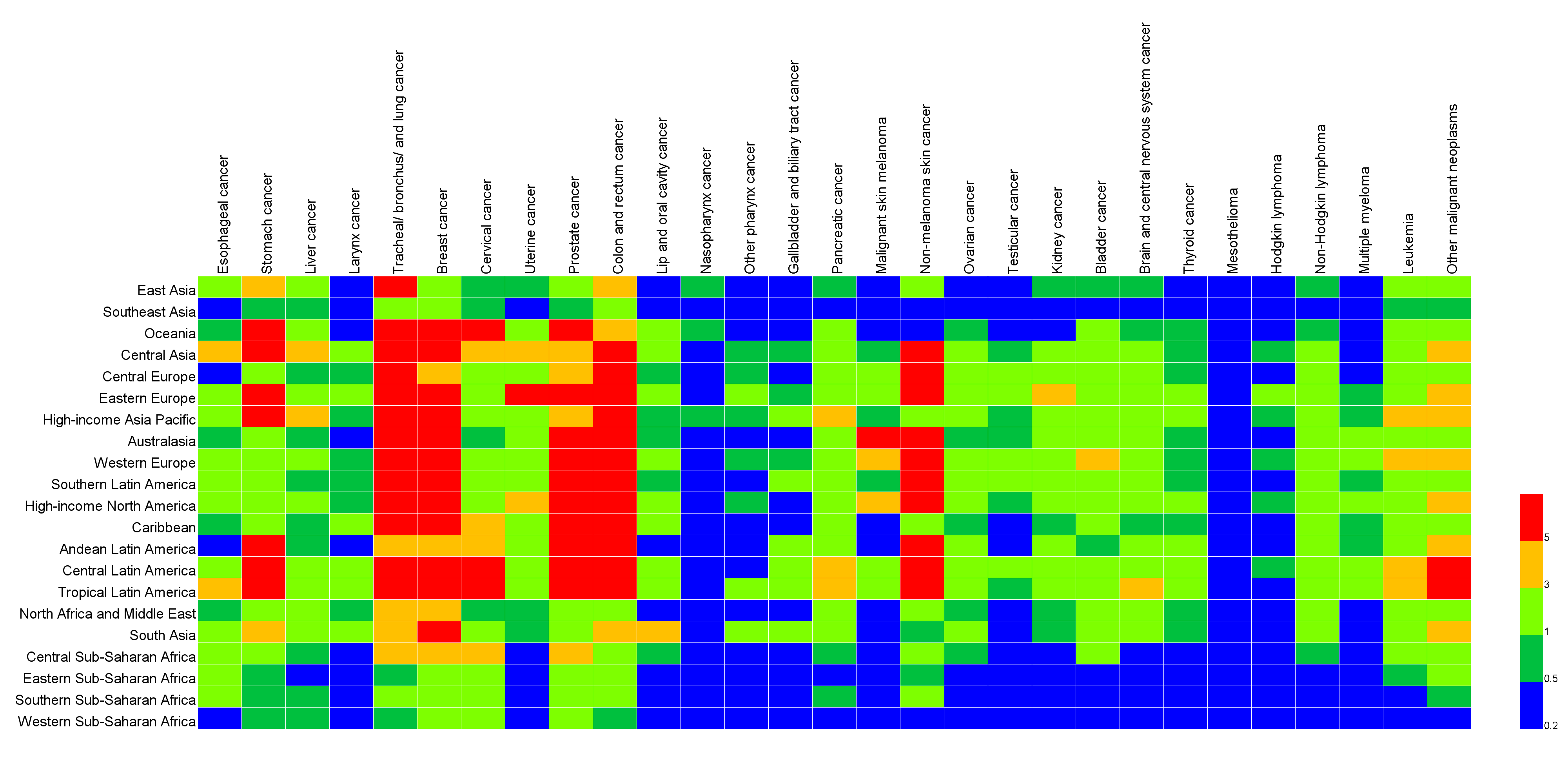


Additional file fig S3. Comparison of the ASDR(A) and ASIR(B) of 29 cancers in 21 regions between 2019 and 1990.


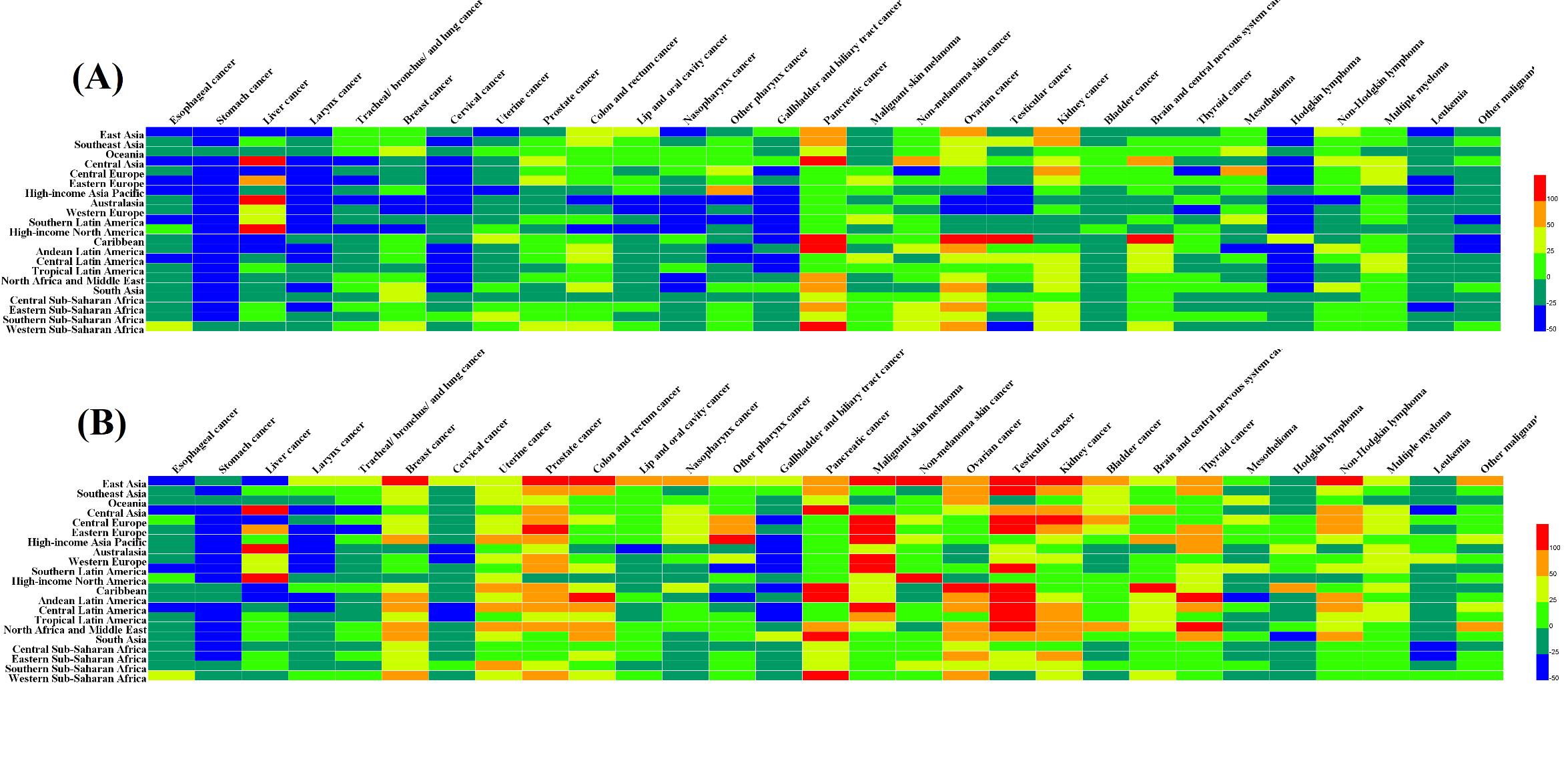


Additional file fig S4. The case fatality rate (CFR) of cancers in different HDI and SDI regions.


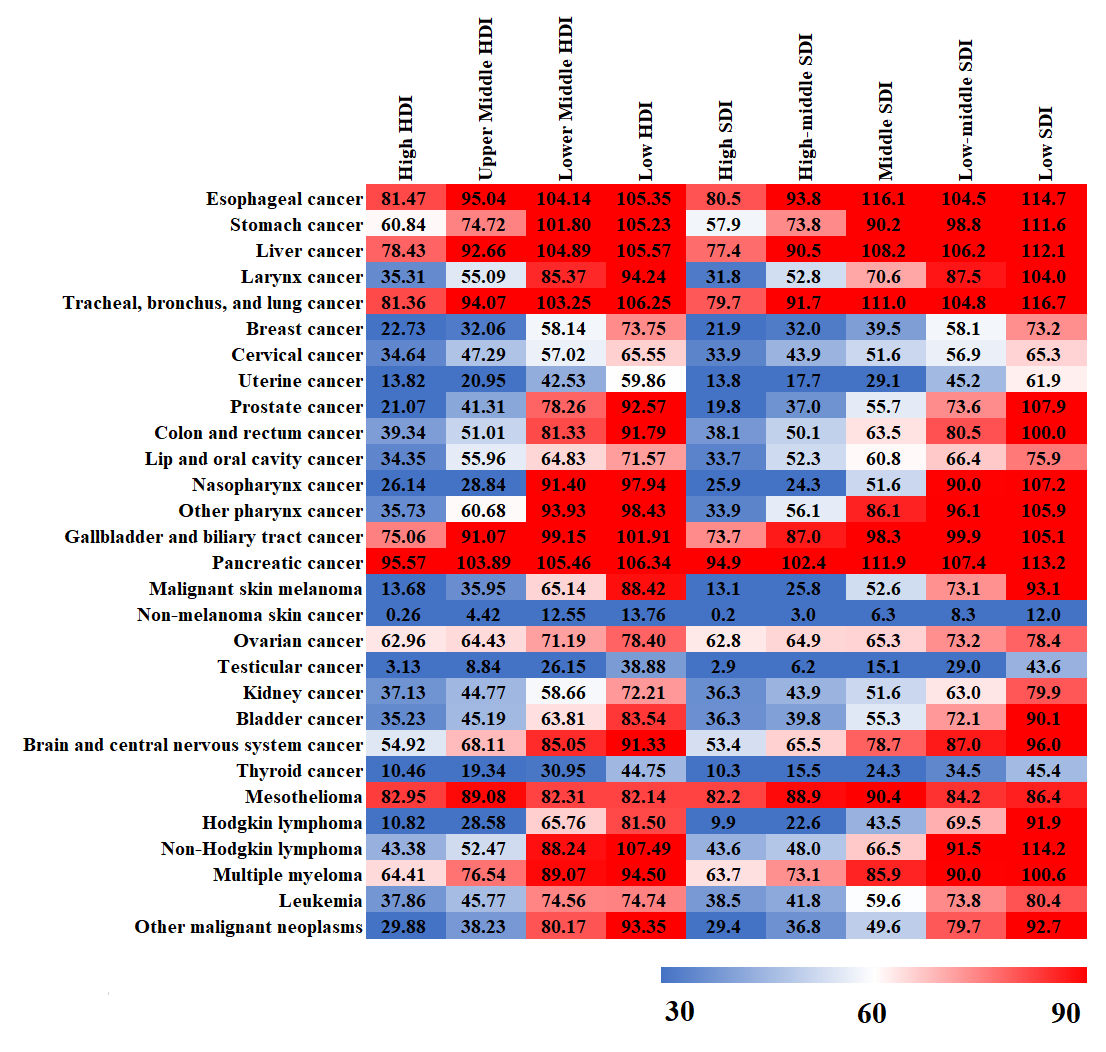


Additional file fig S5. The relative changes in ASDR and ASIR of 29 specified cancer groups between 1990 and 2019.


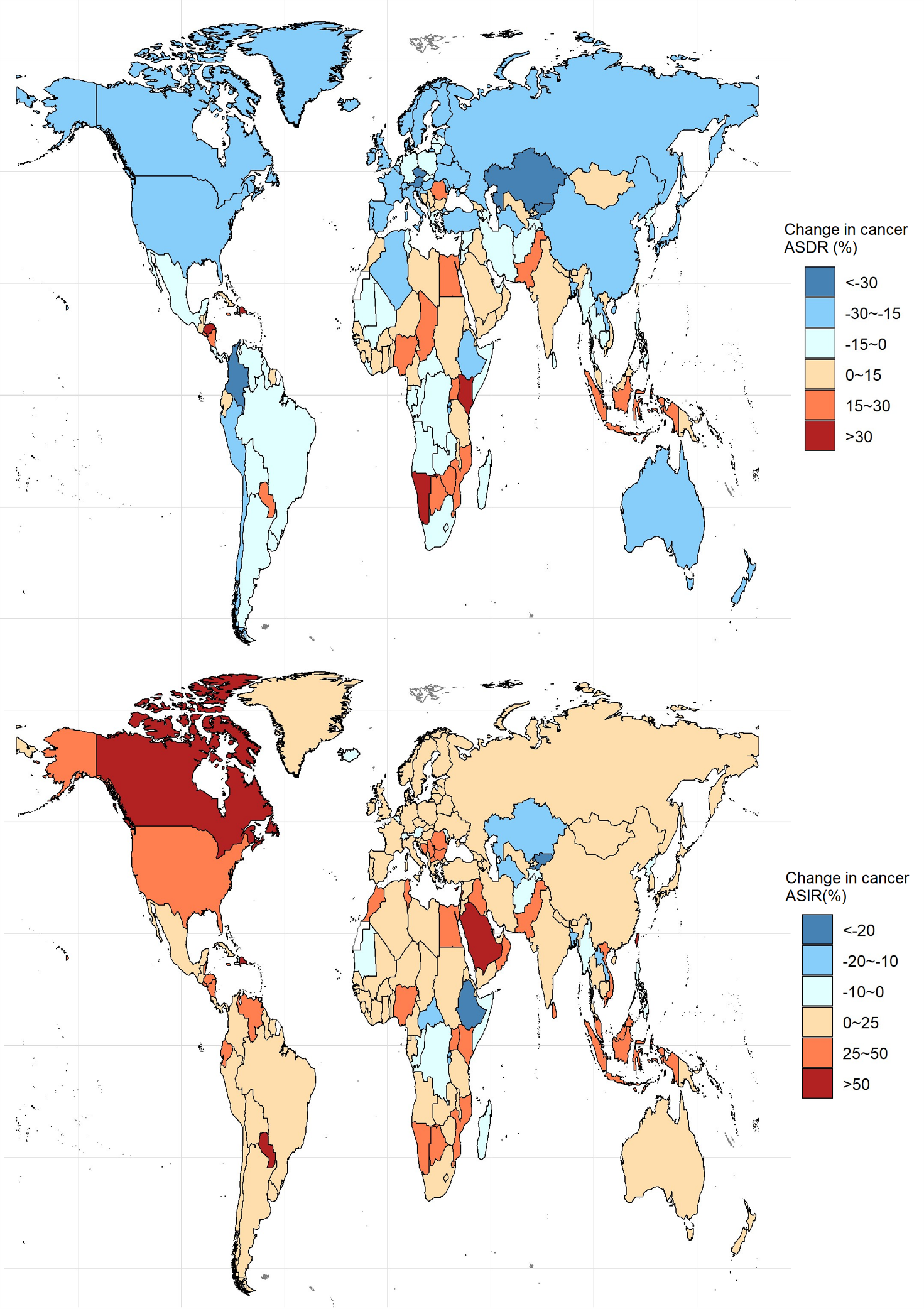


Additional file fig S6. The age-standardized deaths and incidence of cancers in different countries and territories compared with those globally in 2019. (Display the death numbers for the top 50 countries and territories’ results).


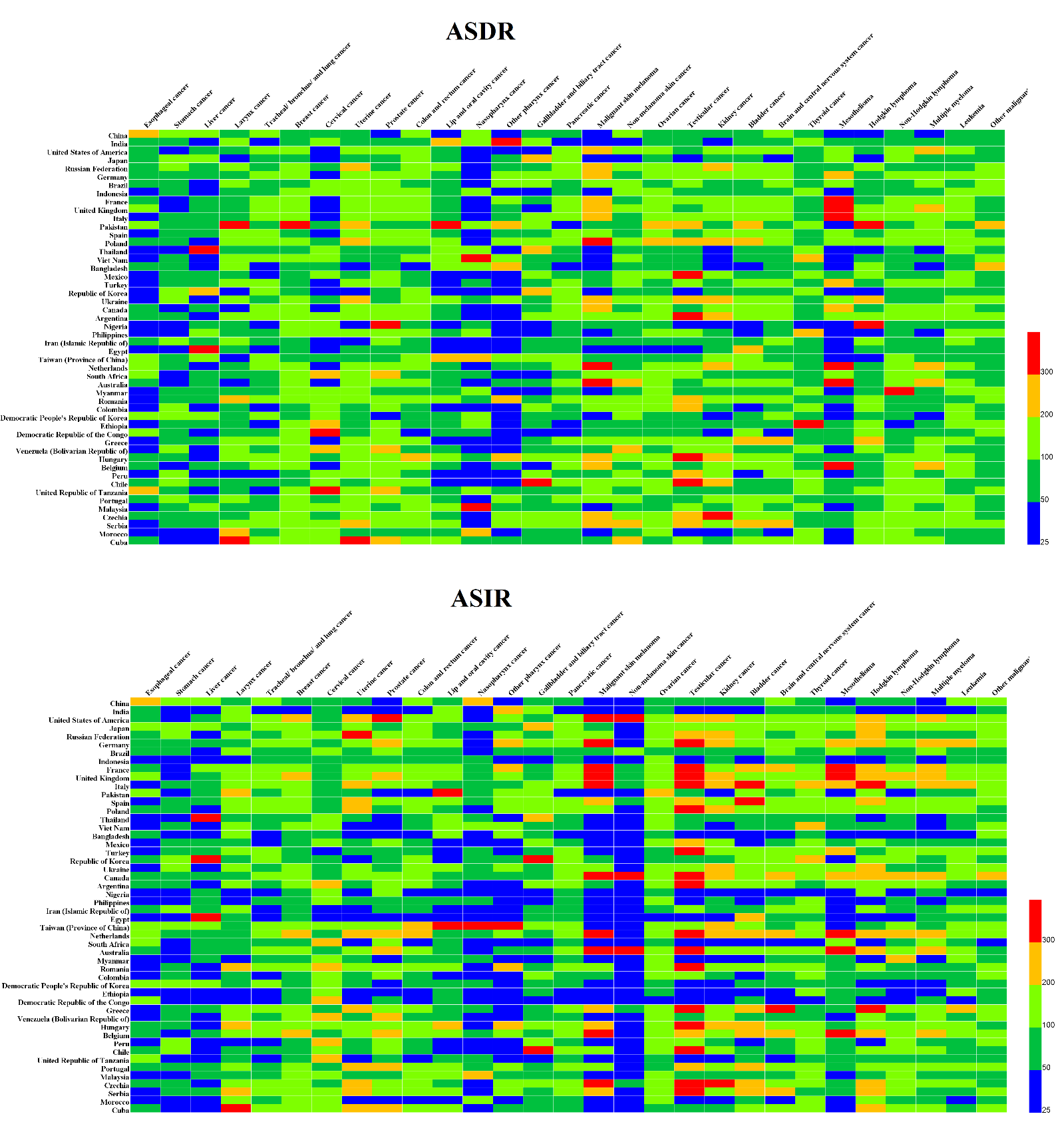


Additional file table S1. Death numbers, incidence number, ASDR, ASIR, and GAL of Pancreatic cancer of 50 countries and territories in 2019.

| **Countries and territories** | **Death number** | **Incidence number** | **ASDR** | **ASIR** | **GAL of ASDR** | **GAL of ASIR** |
| --- | --- | --- | --- | --- | --- | --- |
| global | 531107(566537-491948) | 530297(573635-486175) | 6.62(7.06-6.11) | 6.57(7.09-6) | 100.00 | 100.00 |
| China | 117374(136453-99863) | 114964(133708-98048) | 5.99(6.93-5.12) | 5.78(6.69-4.94) | 90.56 | 88.00 |
| United States of America | 57488(60245-53665) | 58422(67306-50441) | 10.06(10.52-9.43) | 10.37(11.96-8.94) | 152.04 | 157.92 |
| Japan | 37462(40785-31496) | 40981(47875-33125) | 9.6(10.26-8.4) | 10.69(12.47-8.82) | 145.06 | 162.78 |
| India | 33546(38690-29138) | 32482(37343-28160) | 3.13(3.6-2.71) | 2.95(3.4-2.55) | 47.30 | 44.97 |
| Germany | 21508(23693-19347) | 22970(29284-17938) | 10.82(11.83-9.86) | 11.89(15.27-9.28) | 163.49 | 181.09 |
| Russian Federation | 19378(21965-16968) | 18504(21061-16287) | 8.23(9.34-7.2) | 7.88(8.97-6.94) | 124.40 | 120.06 |
| Brazil | 14977(15869-13838) | 14430(15277-13347) | 6.45(6.84-5.94) | 6.16(6.53-5.68) | 97.42 | 93.80 |
| Italy | 14419(15411-12788) | 14686(17334-12030) | 9.59(10.18-8.7) | 10(11.84-8.26) | 144.89 | 152.35 |
| France | 14046(15609-12224) | 13628(17160-10633) | 9.76(10.72-8.72) | 9.78(12.39-7.61) | 147.46 | 148.92 |
| United Kingdom | 12071(12692-11110) | 11862(13881-9899) | 9.22(9.66-8.58) | 9.22(10.83-7.69) | 139.31 | 140.50 |
| Indonesia | 10589(14831-6931) | 10322(14366-6797) | 5.4(7.49-3.51) | 5.04(6.97-3.3) | 81.67 | 76.75 |
| Spain | 7900(8688-6998) | 8035(10038-6254) | 8.04(8.8-7.25) | 8.36(10.47-6.49) | 121.49 | 127.27 |
| Poland | 7447(8799-6269) | 6908(8099-5857) | 10.58(12.52-8.88) | 9.94(11.7-8.41) | 159.86 | 151.37 |
| Republic of Korea | 7303(8150-6519) | 7982(9525-6671) | 8.25(9.2-7.34) | 8.98(10.71-7.51) | 124.64 | 136.83 |
| Turkey | 7132(8754-5729) | 7065(8692-5643) | 8.24(10.08-6.62) | 8.08(9.93-6.47) | 124.47 | 123.12 |
| Mexico | 6853(7853-5866) | 6674(7683-5744) | 6.01(6.87-5.16) | 5.78(6.64-4.97) | 90.78 | 88.08 |
| Argentina | 6513(7109-5940) | 6254(7819-4897) | 11.94(13.02-10.9) | 11.54(14.41-9.02) | 180.37 | 175.70 |
| Canada | 6055(6647-5474) | 6910(8643-5400) | 8.55(9.35-7.76) | 9.82(12.32-7.63) | 129.22 | 149.64 |
| Ukraine | 5949(7009-5022) | 6067(7104-5103) | 8.03(9.42-6.77) | 8.27(9.71-6.92) | 121.28 | 125.99 |
| Thailand | 4141(5331-3096) | 3999(5156-2986) | 4.11(5.3-3.07) | 3.95(5.09-2.96) | 62.12 | 60.18 |
| Viet Nam | 3924(4960-3070) | 3825(4870-2972) | 4.53(5.68-3.57) | 4.3(5.4-3.38) | 68.48 | 65.54 |
| Netherlands | 3913(4307-3517) | 3922(4873-3081) | 11.1(12.16-10.03) | 11.29(14.11-8.83) | 167.68 | 171.89 |
| Romania | 3644(4465-2969) | 3568(4378-2912) | 9.92(12.17-8.11) | 9.86(12.11-8.06) | 149.88 | 150.11 |
| Australia | 3561(3941-3177) | 3779(4730-2929) | 8.26(9.08-7.45) | 8.85(11.1-6.85) | 124.88 | 134.83 |
| Taiwan (Province of China) | 3387(4409-2638) | 3377(4397-2618) | 8.53(11.08-6.64) | 8.54(11.11-6.64) | 128.89 | 130.03 |
| Iran (Islamic Republic of) | 3361(3643-3124) | 3319(3605-3082) | 4.89(5.32-4.51) | 4.72(5.14-4.35) | 73.85 | 71.94 |
| Philippines | 3250(3958-2654) | 3208(3968-2577) | 4.41(5.32-3.64) | 4.19(5.19-3.39) | 66.57 | 63.87 |
| Nigeria | 3217(4014-2468) | 3072(3856-2339) | 4.27(5.25-3.34) | 3.92(4.84-3.03) | 64.47 | 59.67 |
| Pakistan | 3101(3982-2404) | 3033(3955-2376) | 3.1(3.96-2.41) | 2.91(3.76-2.27) | 46.84 | 44.25 |
| Egypt | 2768(3877-1899) | 2801(3942-1918) | 4.43(6.15-3.07) | 4.33(6.01-2.99) | 67.00 | 65.92 |
| Bangladesh | 2734(4080-1714) | 2616(3879-1652) | 2.21(3.3-1.39) | 2.07(3.09-1.3) | 33.44 | 31.52 |
| South Africa | 2628(2955-2348) | 2528(2851-2252) | 6.29(7.04-5.64) | 5.89(6.6-5.26) | 95.07 | 89.67 |
| Czechia | 2530(3053-2044) | 2508(3027-2033) | 11.81(14.29-9.57) | 11.85(14.34-9.59) | 178.51 | 180.42 |
| Colombia | 2496(3167-1920) | 2407(3050-1856) | 4.7(5.98-3.62) | 4.55(5.79-3.51) | 71.04 | 69.29 |
| Greece | 2464(2699-2197) | 2404(3036-1896) | 9.98(10.84-9.03) | 10.03(12.72-7.85) | 150.73 | 152.79 |
| Hungary | 2331(2789-1923) | 2271(2724-1869) | 12.02(14.42-9.9) | 11.88(14.32-9.79) | 181.60 | 180.99 |
| Belgium | 2205(2425-1978) | 2154(2691-1707) | 9.28(10.13-8.45) | 9.28(11.67-7.25) | 140.16 | 141.36 |
| Sweden | 1976(2129-1793) | 1778(2052-1485) | 9.04(9.69-8.31) | 8.4(9.72-7.06) | 136.60 | 127.90 |
| Austria | 1906(2075-1728) | 1964(2384-1604) | 10.34(11.24-9.48) | 10.83(13.17-8.81) | 156.31 | 165.03 |
| Chile | 1874(2057-1689) | 1822(2304-1417) | 7.76(8.53-7) | 7.55(9.55-5.87) | 117.33 | 115.05 |
| Portugal | 1842(2031-1627) | 1681(2138-1310) | 7.44(8.12-6.64) | 7.02(8.99-5.46) | 112.41 | 106.97 |
| Serbia | 1734(2156-1367) | 1631(2038-1283) | 10.8(13.44-8.55) | 10.21(12.76-8.06) | 163.12 | 155.54 |
| Peru | 1722(2250-1303) | 1648(2155-1237) | 5.42(7.08-4.09) | 5.19(6.81-3.9) | 81.95 | 79.02 |
| Myanmar | 1722(2223-1349) | 1666(2162-1299) | 3.95(5.07-3.11) | 3.71(4.79-2.91) | 59.76 | 56.52 |
| Venezuela (Bolivarian Republic of) | 1667(2157-1261) | 1625(2116-1223) | 5.86(7.55-4.45) | 5.65(7.29-4.27) | 88.53 | 86.11 |
| Switzerland | 1581(1742-1406) | 1673(2107-1301) | 8.73(9.59-7.82) | 9.39(11.91-7.28) | 131.85 | 143.06 |
| Bulgaria | 1574(1962-1248) | 1525(1907-1211) | 11.09(13.87-8.78) | 10.93(13.71-8.61) | 167.60 | 166.43 |
| Ghana | 1439(1884-1093) | 1407(1846-1066) | 9.58(12.57-7.34) | 9.07(11.97-6.94) | 144.70 | 138.21 |
| Finland | 1378(1528-1219) | 1486(1874-1172) | 10.71(11.84-9.63) | 11.7(14.78-9.18) | 161.78 | 178.28 |
| Iraq | 1329(1655-1007) | 1333(1668-1003) | 6.12(7.47-4.68) | 5.91(7.26-4.48) | 92.50 | 90.03 |

Additional file table S2. Death numbers, incidence number, ASDR, ASIR, and GAL of TBL cancer of 50 countries and territories in 2019.

| **Countries and territories** | **Death number** | **Incidence number** | **ASDR** | **ASIR** | **GAL of ASDR** | **GAL of ASIR** |
| --- | --- | --- | --- | --- | --- | --- |
| China | 757171(887752-638741) | 832922(981632-700293) | 38.7(45.03-32.8) | 41.71(48.8-35.22) | 153.68 | 150.80 |
| United States of America | 206196(214277-193717) | 254808(294429-220998) | 36.11(37.51-34.09) | 45.13(52.21-39.11) | 143.41 | 163.15 |
| India | 89242(105403-73675) | 87339(103504-71865) | 8.07(9.49-6.65) | 7.7(9.1-6.36) | 32.04 | 27.85 |
| Japan | 86001(92677-73379) | 116798(136541-95776) | 21.21(22.52-18.86) | 30.66(36-25.55) | 84.22 | 110.85 |
| Russian Federation | 54140(63100-46121) | 58184(67802-49721) | 22.77(26.57-19.41) | 24.53(28.58-20.96) | 90.43 | 88.70 |
| Germany | 53968(57287-50506) | 63453(81895-49671) | 29(30.59-27.32) | 35.48(46.13-27.82) | 115.18 | 128.28 |
| Indonesia | 49437(61105-36066) | 48199(59309-35266) | 24.43(30.22-17.87) | 22.87(28.04-16.78) | 97.00 | 82.67 |
| United Kingdom | 42837(44845-39931) | 51490(60654-43156) | 32.85(34.33-30.83) | 40.16(47.33-33.54) | 130.44 | 145.21 |
| France | 40195(42761-37107) | 45064(57492-35106) | 31.53(33.39-29.42) | 36.82(46.99-28.64) | 125.20 | 133.12 |
| Brazil | 36987(38867-34616) | 36410(38236-34234) | 15.79(16.62-14.73) | 15.4(16.17-14.45) | 62.70 | 55.68 |
| Italy | 36975(38886-33918) | 41735(49564-34311) | 25.57(26.72-23.78) | 30.16(36.01-24.64) | 101.56 | 109.02 |
| Poland | 31206(36996-26090) | 30018(35718-25154) | 44.31(52.61-36.96) | 43.06(51.42-35.98) | 175.96 | 155.68 |
| Turkey | 29832(37028-23752) | 29511(36799-23370) | 33.77(41.8-26.89) | 33.08(41.11-26.25) | 134.09 | 119.60 |
| Viet Nam | 25161(31704-19494) | 25550(32387-19741) | 26.98(33.59-21.12) | 26.72(33.51-20.83) | 107.15 | 96.59 |
| Spain | 24523(25958-22753) | 29523(37488-23091) | 26.73(28.23-24.96) | 33.19(42.5-25.86) | 106.14 | 120.00 |
| Canada | 24052(25571-22043) | 30309(38239-23640) | 34.09(36.13-31.42) | 43.63(55.05-33.99) | 135.37 | 157.72 |
| Republic of Korea | 23300(25565-20864) | 31825(37538-26456) | 26.3(28.95-23.51) | 35.61(41.94-29.58) | 104.45 | 128.73 |
| Thailand | 23109(30148-17523) | 22545(29560-17018) | 22.96(29.79-17.39) | 22.24(29.04-16.78) | 91.17 | 80.41 |
| Pakistan | 18550(23969-14209) | 18401(24265-13970) | 17.16(22.05-13.14) | 16.43(21.49-12.47) | 68.15 | 59.41 |
| Ukraine | 17023(20205-14127) | 20133(24383-16536) | 22.79(27-18.87) | 27.19(33.08-22.38) | 90.51 | 98.31 |
| Philippines | 13964(17103-11342) | 13827(17100-11026) | 18.47(22.39-15.13) | 17.71(21.75-14.24) | 73.34 | 64.01 |
| Argentina | 13879(14719-13000) | 13856(17375-10993) | 25.74(27.35-24.14) | 25.89(32.54-20.52) | 102.24 | 93.62 |
| Netherlands | 13163(13990-12162) | 15155(19019-12033) | 38.1(40.47-35.37) | 44.82(56.6-35.35) | 151.28 | 162.03 |
| Taiwan (Province of China) | 12053(15345-9527) | 12618(16206-9963) | 30.35(38.58-24.07) | 31.88(40.93-25.21) | 120.51 | 115.24 |
| Romania | 11014(13362-9116) | 11545(14025-9483) | 31.17(37.83-25.69) | 33.21(40.47-27.24) | 123.76 | 120.07 |
| Mexico | 11002(12698-9425) | 10890(12582-9400) | 9.74(11.24-8.36) | 9.52(10.97-8.23) | 38.69 | 34.41 |
| Myanmar | 10614(14368-7896) | 10291(14071-7608) | 23.8(31.88-17.76) | 22.43(30.17-16.85) | 94.51 | 81.10 |
| Australia | 10034(10723-9207) | 12797(16136-10031) | 23.46(24.97-21.67) | 30.45(38.45-23.87) | 93.16 | 110.07 |
| Bangladesh | 9970(15550-6569) | 9652(15120-6331) | 7.81(12.13-5.19) | 7.43(11.56-4.91) | 31.03 | 26.85 |
| Democratic People's Republic of Korea | 9163(11540-7158) | 9042(11461-7008) | 28.54(35.77-22.5) | 27.88(35.2-21.77) | 113.33 | 100.80 |
| Hungary | 8972(10848-7427) | 9510(11562-7849) | 48.12(58.44-39.58) | 51.94(63.65-42.61) | 191.11 | 187.77 |
| Iran (Islamic Republic of) | 8923(9595-8247) | 8705(9366-8040) | 12.88(13.89-11.86) | 12.24(13.16-11.26) | 51.16 | 44.24 |
| South Africa | 8693(9906-7810) | 8518(9704-7630) | 20(22.58-18.04) | 19.13(21.64-17.2) | 79.42 | 69.14 |
| Greece | 8643(9193-8027) | 9238(11549-7274) | 37.6(39.76-35.3) | 41.98(53.06-32.69) | 149.30 | 151.78 |
| Belgium | 7896(8358-7365) | 8676(10948-6828) | 35.12(37.14-32.93) | 39.84(50.57-31.05) | 139.48 | 144.05 |
| Serbia | 7262(9071-5732) | 7700(9694-6058) | 45.96(57.51-36.13) | 49.38(62.4-38.82) | 182.50 | 178.53 |
| Cuba | 6560(7938-5396) | 6827(8297-5602) | 34.19(41.53-28.1) | 35.88(43.68-29.37) | 135.76 | 129.72 |
| Colombia | 6348(8062-4981) | 6400(8152-5019) | 12(15.26-9.43) | 12.14(15.47-9.52) | 47.65 | 43.90 |
| Czechia | 6238(7580-5137) | 6943(8448-5695) | 29.19(35.52-23.97) | 32.89(40.09-26.85) | 115.90 | 118.91 |
| Nigeria | 6238(8214-4775) | 5887(7545-4570) | 8.27(10.7-6.42) | 7.5(9.46-5.92) | 32.83 | 27.12 |
| Egypt | 6070(8216-4274) | 6123(8313-4303) | 9.5(12.9-6.68) | 9.22(12.46-6.48) | 37.74 | 33.32 |
| Venezuela (Bolivarian Republic of) | 5659(7254-4208) | 5768(7449-4263) | 19.64(25.12-14.69) | 19.78(25.4-14.72) | 77.98 | 71.49 |
| Morocco | 5337(6881-3834) | 5277(6807-3793) | 16.98(21.74-12.17) | 16.36(20.97-11.74) | 67.44 | 59.13 |
| Malaysia | 5221(6639-4060) | 5165(6561-3997) | 20.6(25.95-16.01) | 19.78(25.12-15.37) | 81.80 | 71.52 |
| Portugal | 4709(5015-4367) | 4680(5919-3656) | 20.74(22.05-19.33) | 21.38(27.28-16.53) | 82.37 | 77.28 |
| Bulgaria | 4608(5714-3701) | 4838(6016-3859) | 34.03(42.61-26.93) | 36.36(45.51-28.73) | 135.15 | 131.44 |
| Denmark | 4359(4718-4014) | 4932(6075-3887) | 37(40.02-34.15) | 42.91(53.14-33.7) | 146.93 | 155.12 |
| Sweden | 4337(4594-4013) | 4342(5018-3695) | 20.04(21.16-18.72) | 20.78(24.16-17.67) | 79.57 | 75.14 |
| Democratic Republic of the Congo | 4314(8647-2287) | 4254(8568-2251) | 12.52(24.38-6.6) | 11.87(23.34-6.26) | 49.72 | 42.90 |
| Iraq | 4232(5190-3275) | 4154(5129-3200) | 19.86(23.82-15.62) | 18.69(22.68-14.53) | 78.87 | 67.58 |

Additional file table S3. Death numbers, incidence number, ASDR, ASIR, and GAL of females breast cancer of 50 countries and territories in 2019.

| **Countries and territories** | **Death number** | **Incidence number** | **ASDR** | **ASIR** | **GAL of ASDR** | **GAL of ASIR** |
| --- | --- | --- | --- | --- | --- | --- |
| China | 93499(115420-74511) | 368375(463336-290086) | 15.88(17.07-14.66) | 45.86(49.76-41.91) | 56.76 | 77.66 |
| India | 82099(104727-63114) | 144086(181150-110434) | 9.02(11.1-7.19) | 35.61(44.81-28.07) | 86.06 | 50.23 |
| United States of America | 54402(57271-50420) | 251531(305346-207355) | 13.67(17.35-10.57) | 23.04(28.97-17.79) | 115.83 | 205.44 |
| Pakistan | 31177(42602-23333) | 50293(68299-36575) | 18.4(19.23-17.34) | 94.21(115.12-77.35) | 327.00 | 166.81 |
| Indonesia | 26166(33676-20171) | 50628(66656-38906) | 51.94(69.76-39.03) | 76.49(102.5-56.07) | 128.89 | 81.61 |
| Russian Federation | 23486(27969-19329) | 67693(82510-55292) | 20.47(25.94-15.89) | 37.42(48.59-28.96) | 107.15 | 118.61 |
| Germany | 21486(23348-19235) | 69662(89596-53510) | 17.02(20.46-13.91) | 54.39(66.87-44.01) | 132.45 | 184.70 |
| Brazil | 19764(21001-18415) | 51856(55276-48535) | 21.04(22.52-19.19) | 84.7(111.39-64.8) | 95.23 | 86.45 |
| Japan | 15911(17288-13374) | 74260(90978-59292) | 15.13(16.08-14.1) | 39.64(42.28-37.1) | 63.85 | 131.87 |
| United Kingdom | 15162(16011-13811) | 52730(65672-41949) | 10.14(10.78-9.24) | 60.47(74.56-48.35) | 142.15 | 205.61 |
| France | 15138(16690-12908) | 50715(65459-39237) | 22.58(23.63-21.01) | 94.29(118.81-74.32) | 125.56 | 189.51 |
| Italy | 14309(15346-12454) | 53095(67752-40906) | 19.94(21.53-17.94) | 86.9(113.15-66.2) | 116.27 | 194.46 |
| Nigeria | 13331(18430-9350) | 21121(29478-14363) | 18.47(19.6-16.8) | 89.17(114.87-68.53) | 169.89 | 84.67 |
| Viet Nam | 11732(15223-8927) | 27432(35854-20526) | 26.98(36.34-19.48) | 38.82(52.89-27) | 135.58 | 106.09 |
| Philippines | 10110(13135-7604) | 19032(24890-14207) | 21.54(27.78-16.39) | 48.65(63.02-36.57) | 143.84 | 88.59 |
| Bangladesh | 9771(12280-7661) | 17661(22672-13693) | 22.85(29.48-17.39) | 40.63(52.96-30.52) | 91.54 | 54.59 |
| Ukraine | 8473(10581-6708) | 17667(22279-13772) | 14.54(18.15-11.51) | 25.03(31.81-19.56) | 124.49 | 98.22 |
| Argentina | 8403(9066-7742) | 17745(22569-13747) | 19.77(24.92-15.57) | 45.04(57.25-35.01) | 175.76 | 136.77 |
| Mexico | 8024(9777-6649) | 24312(29810-19777) | 27.92(30.04-25.85) | 62.72(80.34-48.36) | 78.58 | 80.27 |
| Spain | 7981(8763-7002) | 29411(37841-22819) | 12.48(15.16-10.35) | 36.81(45.04-30.02) | 95.43 | 152.43 |
| Poland | 7491(9292-5993) | 18544(23330-14430) | 15.16(16.34-13.79) | 69.9(90.54-53.57) | 120.88 | 121.11 |
| Thailand | 6790(8937-4986) | 17657(23719-12731) | 19.2(24.04-15.34) | 55.54(70.93-43.24) | 78.13 | 72.65 |
| Canada | 6515(7113-5843) | 28469(36022-22222) | 12.41(16.35-9.08) | 33.31(44.6-23.94) | 113.33 | 196.48 |
| Turkey | 5926(7337-4729) | 17130(21566-13440) | 18(19.52-16.38) | 90.1(115.61-70.12) | 79.63 | 78.69 |
| South Africa | 5209(5915-4576) | 8540(9820-7370) | 12.65(15.67-10.07) | 36.08(45.46-28.33) | 128.93 | 69.96 |
| Iran (Islamic Republic of) | 4704(5192-4306) | 14743(16469-13248) | 20.48(23.22-18.08) | 32.08(36.95-27.82) | 74.65 | 74.25 |
| Egypt | 4650(6344-3159) | 10600(14525-7356) | 11.86(13.1-10.82) | 34.05(37.94-30.69) | 89.59 | 63.92 |
| Democratic Republic of the Congo | 4612(6237-3262) | 6415(8717-4506) | 14.23(19.18-9.59) | 29.31(39.96-20.1) | 141.79 | 62.76 |
| Myanmar | 4556(5721-3731) | 7540(9563-5992) | 22.52(30.58-15.82) | 28.78(39.45-20.24) | 105.64 | 58.09 |
| Morocco | 4372(5942-3195) | 9755(13518-7043) | 16.78(20.84-13.87) | 26.64(33.54-21.39) | 153.67 | 114.52 |
| Netherlands | 4320(4729-3865) | 16608(21207-12844) | 24.41(32.79-18.06) | 52.52(71.97-38.19) | 151.93 | 243.13 |
| Republic of Korea | 4063(4548-3582) | 20251(24449-16357) | 24.13(26.1-22.03) | 111.49(143.86-85.92) | 54.93 | 101.18 |
| Ethiopia | 3702(4509-2966) | 5446(6858-4211) | 8.73(9.73-7.71) | 46.4(56.14-37.59) | 109.29 | 50.16 |
| Australia | 17.09(18.43-15.55) | 81.55(105.51-62.85) | 17.36(20.5-14.3) | 23(28.15-18.38) | 107.62 | 177.83 |
| Romania | 18.47(22.58-15.07) | 50.92(62.49-41.05) | 17.09(18.43-15.55) | 81.55(105.51-62.85) | 116.31 | 111.04 |
| Malaysia | 25.81(32.54-19.83) | 59.48(75.15-45.19) | 18.47(22.58-15.07) | 50.92(62.49-41.05) | 162.51 | 129.71 |
| Colombia | 11.71(14.95-9.12) | 39.41(50.8-30.06) | 25.81(32.54-19.83) | 59.48(75.15-45.19) | 73.70 | 85.94 |
| Iraq | 21.64(28.07-16.4) | 52(68.9-38.87) | 11.71(14.95-9.12) | 39.41(50.8-30.06) | 136.23 | 113.40 |
| Belgium | 23.09(24.94-21.08) | 95.21(123.57-73.85) | 21.64(28.07-16.4) | 52(68.9-38.87) | 145.36 | 207.63 |
| Ghana | 30.32(38.21-24.07) | 45.06(57.59-35.11) | 23.09(24.94-21.08) | 95.21(123.57-73.85) | 190.88 | 98.27 |
| Greece | 21.65(23.33-19.71) | 85.49(111.32-65.14) | 30.32(38.21-24.07) | 45.06(57.59-35.11) | 136.27 | 186.43 |
| Venezuela (Bolivarian Republic of) | 17.65(23.06-13.43) | 53(70.51-39.43) | 21.65(23.33-19.71) | 85.49(111.32-65.14) | 111.13 | 115.57 |
| Algeria | 13.86(17.32-10.66) | 34(43.29-25.64) | 17.65(23.06-13.43) | 53(70.51-39.43) | 87.25 | 74.14 |
| United Republic of Tanzania | 17.84(21.43-14.24) | 23.86(29.23-18.68) | 13.86(17.32-10.66) | 34(43.29-25.64) | 112.34 | 52.02 |
| Serbia | 29.1(36.16-23.19) | 83.48(107.23-64.76) | 17.84(21.43-14.24) | 23.86(29.23-18.68) | 183.21 | 182.03 |
| Democratic People's Republic of Korea | 13(17.37-9.49) | 26.56(37.11-18.5) | 29.1(36.16-23.19) | 83.48(107.23-64.76) | 81.82 | 57.92 |
| Taiwan (Province of China) | 11.57(15-9.03) | 49.42(65.91-37.24) | 13(17.37-9.49) | 26.56(37.11-18.5) | 72.85 | 107.77 |
| Uzbekistan | 17.43(20.62-14.55) | 37.27(44.82-30.57) | 11.57(15-9.03) | 49.42(65.91-37.24) | 109.71 | 81.27 |
| Nepal | 18.08(23.83-13.41) | 28.81(38.35-21.2) | 17.43(20.62-14.55) | 37.27(44.82-30.57) | 113.85 | 62.84 |
| Hungary | 20.06(24.23-16.31) | 64(79.08-51.13) | 18.08(23.83-13.41) | 28.81(38.35-21.2) | 126.30 | 139.56 |

Additional file table S4. Death numbers, incidence number, ASDR, ASIR, CFR, and GAL of leukemia of 50 countries and territories in 2019.

| **Countries and territories** | **Death number** | **Incidence number** | **ASDR** | **ASIR** | **CFR(%)** | **ASDR-GAL** | **ASIR-GAL** |
| --- | --- | --- | --- | --- | --- | --- | --- |
| China | 60381(70988-50213) | 154648(181075-127207) | 3.67(4.28-3.07) | 10.47(12.34-8.7) | 35.04 | 86.02 | 127.31 |
| India | 33827(40416-28764) | 43570(52165-37235) | 2.87(3.42-2.44) | 3.56(4.25-3.03) | 80.5 | 67.22 | 43.3 |
| United States of America | 31378(33016-28944) | 53107(61437-45610) | 5.74(6.01-5.36) | 10.2(11.77-8.88) | 56.24 | 134.63 | 124.12 |
| Indonesia | 10591(12911-8501) | 15269(18468-12252) | 4.93(5.95-3.99) | 6.71(8.08-5.41) | 73.46 | 115.57 | 81.57 |
| Japan | 9860(10580-8419) | 22630(26390-19076) | 3.09(3.25-2.79) | 10.81(12.49-9.34) | 28.61 | 72.54 | 131.46 |
| Germany | 9336(10278-8160) | 32361(41856-25105) | 4.88(5.31-4.38) | 21.91(28.45-17.17) | 22.28 | 114.52 | 266.49 |
| Brazil | 8796(9217-8199) | 11994(12642-11229) | 3.96(4.16-3.68) | 5.52(5.86-5.13) | 71.75 | 92.93 | 67.16 |
| France | 7698(8584-6499) | 17596(21914-13872) | 5.32(5.82-4.7) | 16.08(20.3-12.74) | 33.06 | 124.72 | 195.64 |
| Russian Federation | 7427(8468-6454) | 14266(16317-12502) | 3.55(4.03-3.11) | 7.09(8.06-6.29) | 50.1 | 83.32 | 86.24 |
| Italy | 7153(7633-6290) | 20461(24399-17021) | 5.1(5.38-4.61) | 20.87(24.82-17.47) | 24.43 | 119.58 | 253.81 |
| Pakistan | 6492(7817-5336) | 10833(13875-8451) | 3.94(4.72-3.28) | 5.48(6.77-4.49) | 71.87 | 92.44 | 66.7 |
| United Kingdom | 5857(6139-5317) | 12648(14965-10623) | 4.67(4.87-4.33) | 12.55(14.72-10.66) | 37.25 | 109.61 | 152.59 |
| Ethiopia | 5265(7254-2912) | 8308(12435-4274) | 7.74(10.92-4.51) | 9.5(13.36-5.31) | 81.47 | 181.57 | 115.56 |
| Mexico | 5249(5958-4603) | 7787(8825-6810) | 4.37(4.95-3.84) | 6.44(7.28-5.63) | 67.93 | 102.59 | 78.31 |
| Iran (Islamic Republic of) | 4424(5030-3137) | 6767(7890-4646) | 6.04(6.85-4.31) | 8.85(10.29-6.17) | 68.25 | 141.76 | 107.7 |
| Turkey | 4405(5642-3490) | 6884(8616-5516) | 5.28(6.68-4.21) | 8.45(10.48-6.84) | 62.43 | 123.75 | 102.78 |
| Philippines | 4213(4971-3539) | 6954(8425-5838) | 4.55(5.42-3.81) | 6.79(8.2-5.71) | 67.06 | 106.77 | 82.56 |
| Spain | 4191(4629-3707) | 10958(13779-8616) | 4.42(4.78-4.01) | 16.1(20.33-12.74) | 27.47 | 103.73 | 195.79 |
| Thailand | 4143(5700-2482) | 6078(8201-3951) | 4.69(6.35-2.95) | 8.04(10.46-5.46) | 58.27 | 109.93 | 97.83 |
| Nigeria | 3503(4614-2603) | 5200(6947-3704) | 2.87(3.58-2.24) | 3.46(4.39-2.7) | 82.76 | 67.24 | 42.13 |
| Bangladesh | 3495(4647-2544) | 4535(5952-3361) | 2.55(3.39-1.86) | 3.2(4.19-2.38) | 79.84 | 59.85 | 38.87 |
| Poland | 3451(4042-2931) | 6333(7511-5328) | 5.21(6.07-4.43) | 10.3(12.17-8.75) | 50.53 | 122.11 | 125.3 |
| Viet Nam | 3391(4279-2575) | 4525(5834-3431) | 3.81(4.83-2.95) | 4.95(6.34-3.81) | 76.98 | 89.39 | 60.21 |
| Canada | 3325(3635-2977) | 8438(10626-6506) | 4.96(5.38-4.5) | 15.1(19.02-11.69) | 32.81 | 116.23 | 183.67 |
| Ukraine | 2950(3532-2499) | 4959(5860-4176) | 4.78(5.69-4.07) | 8.6(10-7.33) | 55.63 | 112.24 | 104.62 |
| Egypt | 2806(3774-2034) | 4068(5604-2930) | 3.78(5.33-2.63) | 4.98(6.83-3.55) | 75.99 | 88.75 | 60.56 |
| Myanmar | 2659(3469-1965) | 4219(5833-2930) | 5.53(7.15-4.19) | 8.36(11.44-5.93) | 66.16 | 129.81 | 101.75 |
| Argentina | 2658(2839-2460) | 3516(4372-2787) | 5.16(5.5-4.8) | 7.1(8.83-5.63) | 72.74 | 121.1 | 86.33 |
| Colombia | 2186(2744-1704) | 3373(4224-2622) | 4.39(5.5-3.42) | 6.98(8.73-5.43) | 62.94 | 103.04 | 84.9 |
| Afghanistan | 2120(3185-1410) | 3360(5172-2125) | 10.01(14.38-6.95) | 12.36(18.15-8.39) | 80.95 | 234.74 | 150.36 |
| Republic of Korea | 2049(2393-1514) | 5983(7467-4174) | 2.69(3.15-2) | 9.19(11.45-6.7) | 29.28 | 63.14 | 111.83 |
| Australia | 1986(2174-1763) | 3883(4909-2993) | 4.79(5.18-4.3) | 10.08(12.72-7.82) | 47.48 | 112.29 | 122.64 |
| Iraq | 1876(2351-1453) | 3175(3968-2441) | 6.95(8.75-5.4) | 9.89(12.3-7.69) | 70.32 | 163.15 | 120.3 |
| Syrian Arab Republic | 1847(2431-1377) | 2665(3501-2014) | 15.82(20.41-11.96) | 20.88(27.37-15.92) | 75.79 | 371.14 | 253.92 |
| Netherlands | 1833(2010-1624) | 3967(5002-3110) | 5.43(5.9-4.87) | 15.3(19.31-11.93) | 35.48 | 127.34 | 186.1 |
| Sudan | 1685(2346-1127) | 2837(4045-1892) | 6.44(8.69-4.4) | 8.76(12.03-5.9) | 73.58 | 151.17 | 106.53 |
| United Republic of Tanzania | 1647(2421-1140) | 3486(5504-2200) | 3.65(4.85-2.73) | 5.69(8.19-4.06) | 64.15 | 85.63 | 69.21 |
| Greece | 1631(1768-1436) | 3269(4045-2598) | 6.59(7.05-5.95) | 17.73(22.23-13.86) | 37.15 | 154.51 | 215.68 |
| Democratic Republic of the Congo | 1498(1984-1064) | 2488(3471-1785) | 2.81(3.96-1.91) | 3.61(4.84-2.55) | 77.86 | 65.98 | 43.94 |
| South Africa | 1494(1683-1222) | 2092(2373-1780) | 3.34(3.76-2.66) | 4.37(4.94-3.66) | 76.36 | 78.34 | 53.2 |
| Peru | 1453(1985-998) | 2185(3034-1453) | 4.4(6-3.03) | 6.59(9.16-4.38) | 66.77 | 103.29 | 80.22 |
| Malaysia | 1391(1805-1098) | 2003(2613-1568) | 5.1(6.64-4.01) | 7.05(9.18-5.52) | 72.31 | 119.56 | 85.74 |
| Romania | 1334(1611-1099) | 2134(2580-1753) | 4.16(4.97-3.43) | 7.36(8.78-6.09) | 56.46 | 97.53 | 89.58 |
| Democratic People's Republic of Korea | 1312(1725-980) | 2036(2652-1536) | 4.49(5.81-3.4) | 7.6(9.85-5.77) | 59.13 | 105.44 | 92.47 |
| Taiwan (Province of China) | 1290(1656-1012) | 2675(3447-2056) | 3.76(4.79-2.99) | 9.26(11.86-7.25) | 40.57 | 88.09 | 112.59 |
| Belgium | 1238(1362-1076) | 2626(3296-2069) | 5.26(5.72-4.73) | 13.97(17.54-10.95) | 37.66 | 123.45 | 169.97 |
| Yemen | 1203(1735-796) | 1892(2740-1243) | 6.57(9.14-4.47) | 8.32(11.91-5.6) | 79.02 | 154.21 | 101.2 |
| Venezuela (Bolivarian Republic of) | 1155(1493-891) | 1633(2097-1245) | 4.13(5.32-3.21) | 5.84(7.5-4.46) | 70.84 | 96.97 | 70.98 |
| Uzbekistan | 1103(1312-939) | 1846(2177-1564) | 4.24(4.93-3.66) | 6.3(7.34-5.4) | 67.32 | 99.48 | 76.63 |
| Portugal | 1072(1176-951) | 2405(3016-1865) | 4.65(5.03-4.19) | 13.54(17.03-10.34) | 34.33 | 109.07 | 164.74 |

Additional file table S5. Death numbers, incidence number, ASDR, ASIR, and GAL of esophageal cancer of 50 countries and territories in 2019.

| **Countries and territories** | **Death number** | **Incidence number** | **ASDR** | **ASIR** | **GAL of ASDR** | **GAL of ASIR** |
| --- | --- | --- | --- | --- | --- | --- |
| China | 257316(309029-202777) | 278121(331600-213512) | 13.15(15.68-10.27) | 13.9(16.52-10.7) | 215.25 | 213.54 |
| India | 38981(56283-32203) | 38516(55988-31813) | 3.49(5.05-2.9) | 3.36(4.89-2.78) | 57.18 | 51.62 |
| United States of America | 21615(22521-20554) | 23150(27406-19583) | 3.86(4.02-3.69) | 4.2(4.98-3.54) | 63.21 | 64.48 |
| Japan | 14000(15103-12469) | 21521(25788-17654) | 3.91(4.19-3.59) | 6.45(7.79-5.29) | 64.04 | 99.09 |
| Brazil | 12551(13222-11802) | 12473(13087-11792) | 5.28(5.56-4.95) | 5.2(5.45-4.9) | 86.39 | 79.86 |
| United Kingdom | 10037(10481-9366) | 10341(12395-8560) | 7.77(8.09-7.31) | 8.23(9.88-6.8) | 127.27 | 126.51 |
| Pakistan | 8953(11011-7184) | 8911(10991-7068) | 8.23(9.96-6.62) | 7.86(9.63-6.3) | 134.75 | 120.81 |
| Russian Federation | 7663(8935-6457) | 7953(9370-6736) | 3.24(3.79-2.73) | 3.38(3.98-2.86) | 53.11 | 51.92 |
| Germany | 6746(7413-6150) | 9602(12502-7438) | 3.71(4.07-3.39) | 5.48(7.2-4.24) | 60.81 | 84.13 |
| France | 5216(5765-4672) | 5813(7406-4539) | 3.96(4.35-3.55) | 4.62(5.95-3.58) | 64.79 | 71.04 |
| Bangladesh | 5110(7680-3629) | 4982(7507-3522) | 4(6-2.87) | 3.81(5.73-2.71) | 65.47 | 58.62 |
| Indonesia | 5069(7233-4062) | 4989(7218-3956) | 2.51(3.58-2.02) | 2.36(3.41-1.89) | 41.07 | 36.28 |
| South Africa | 4508(5540-4008) | 4364(5453-3860) | 10.57(12.54-9.46) | 9.91(12.09-8.78) | 172.97 | 152.34 |
| Thailand | 3096(4168-2001) | 3246(4396-2069) | 3(4.03-1.95) | 3.13(4.22-2.01) | 49.15 | 48.07 |
| Taiwan (Province of China) | 3086(4087-2342) | 3864(5131-2922) | 7.89(10.38-6.02) | 9.99(13.27-7.58) | 129.22 | 153.45 |
| Iran (Islamic Republic of) | 3069(3361-2420) | 3031(3332-2417) | 4.54(4.99-3.54) | 4.36(4.8-3.43) | 74.28 | 66.99 |
| United Republic of Tanzania | 2986(4214-1941) | 2910(4150-1881) | 12.56(17.39-8.13) | 11.85(16.53-7.69) | 205.61 | 182.03 |
| Democratic Republic of the Congo | 2938(4244-1560) | 2884(4156-1530) | 8.71(12.77-4.6) | 8.17(11.95-4.29) | 142.57 | 125.5 |
| Democratic People's Republic of Korea | 2906(3752-2259) | 2923(3771-2253) | 9.01(11.56-7.06) | 8.96(11.6-6.96) | 147.47 | 137.72 |
| Kenya | 2831(4035-2156) | 2576(3623-1935) | 13.72(19.58-10.54) | 11.93(16.74-8.96) | 224.55 | 183.32 |
| Argentina | 2759(3007-2527) | 2681(3366-2121) | 5.08(5.53-4.66) | 4.97(6.25-3.93) | 83.16 | 76.44 |
| Viet Nam | 2727(3470-1899) | 2824(3620-1926) | 2.87(3.58-2.07) | 2.89(3.65-2.04) | 46.94 | 44.47 |
| Italy | 2635(2829-2394) | 2812(3365-2286) | 1.83(1.96-1.69) | 2.05(2.48-1.67) | 30.02 | 31.53 |
| Canada | 2527(2763-2265) | 3002(3792-2347) | 3.64(3.98-3.27) | 4.42(5.59-3.44) | 59.53 | 67.94 |
| Netherlands | 2399(2638-2164) | 2810(3531-2175) | 6.98(7.64-6.34) | 8.37(10.58-6.47) | 114.2 | 128.66 |
| Spain | 2330(2571-2105) | 2656(3391-2048) | 2.55(2.81-2.32) | 3.03(3.9-2.31) | 41.78 | 46.5 |
| Uganda | 2245(2837-1719) | 2203(2805-1674) | 16.53(20.6-12.84) | 15.61(19.47-12.06) | 270.68 | 239.9 |
| Poland | 2233(2706-1834) | 2144(2610-1754) | 3.28(3.99-2.7) | 3.19(3.91-2.6) | 53.71 | 49.09 |
| Republic of Korea | 2193(3173-1891) | 3419(4689-2691) | 2.43(3.52-2.09) | 3.76(5.16-2.96) | 39.72 | 57.78 |
| Ukraine | 2033(2494-1612) | 2163(2696-1724) | 2.78(3.42-2.2) | 2.99(3.72-2.39) | 45.54 | 45.87 |
| Malawi | 1843(2468-1382) | 1810(2438-1346) | 25.76(33.94-19.76) | 24.53(32.51-18.74) | 421.75 | 376.91 |
| Mexico | 1720(2008-1456) | 1668(1962-1417) | 1.51(1.75-1.28) | 1.44(1.69-1.23) | 24.73 | 22.18 |
| Australia | 1720(1905-1525) | 1815(2342-1396) | 4.03(4.45-3.61) | 4.34(5.61-3.34) | 66.05 | 66.69 |
| Turkey | 1333(1682-1032) | 1380(1734-1071) | 1.52(1.93-1.18) | 1.56(1.96-1.21) | 24.92 | 23.91 |
| Uzbekistan | 1320(1562-1084) | 1328(1575-1088) | 6.98(8.09-5.88) | 6.58(7.66-5.51) | 114.3 | 101.06 |
| Kazakhstan | 1276(1482-1088) | 1238(1447-1052) | 7.8(9.02-6.67) | 7.35(8.51-6.27) | 127.66 | 112.99 |
| Ethiopia | 1119(1569-869) | 1079(1505-852) | 2.87(4.03-2.24) | 2.67(3.72-2.11) | 46.96 | 41.03 |
| Sri Lanka | 1115(1502-823) | 1170(1570-853) | 4.49(6.01-3.32) | 4.61(6.19-3.4) | 73.57 | 70.91 |
| Madagascar | 1108(1557-698) | 1100(1557-689) | 10.31(14.36-6.61) | 9.74(13.64-6.18) | 168.76 | 149.69 |
| Nepal | 1091(1480-804) | 1053(1428-772) | 5.1(6.8-3.81) | 4.78(6.48-3.55) | 83.49 | 73.43 |
| Zimbabwe | 1087(1348-853) | 1084(1346-843) | 16.02(19.64-12.75) | 15.4(18.95-12.22) | 262.3 | 236.57 |
| Colombia | 1065(1372-812) | 1013(1308-767) | 2(2.58-1.52) | 1.91(2.47-1.44) | 32.73 | 29.38 |
| Myanmar | 1060(1604-839) | 1039(1577-816) | 2.37(3.58-1.9) | 2.25(3.39-1.79) | 38.77 | 34.58 |
| Philippines | 1046(1418-840) | 1052(1402-837) | 1.36(1.88-1.1) | 1.32(1.75-1.06) | 22.33 | 20.33 |
| Belgium | 1007(1101-913) | 1129(1432-883) | 4.52(4.93-4.12) | 5.26(6.68-4.04) | 73.92 | 80.78 |
| Zambia | 975(1330-635) | 966(1329-624) | 14.83(19.76-9.78) | 14.1(18.84-9.26) | 242.86 | 216.65 |
| Egypt | 968(1286-661) | 987(1318-670) | 1.54(2.09-1.06) | 1.5(2.01-1.04) | 25.15 | 23.03 |
| Cuba | 966(1191-770) | 985(1219-783) | 5.08(6.28-4.05) | 5.21(6.46-4.14) | 83.14 | 80.12 |
| Chile | 964(1065-854) | 935(1178-728) | 4(4.42-3.54) | 3.87(4.89-3.02) | 65.46 | 59.52 |
| Angola | 920(1238-502) | 908(1221-498) | 8.81(11.89-4.73) | 8.24(11.1-4.44) | 144.28 | 126.62 |
